# Higher angiotensin-converting enzyme 2 (ACE2) levels in the brain of individuals with Alzheimer’s disease

**DOI:** 10.1186/s40478-023-01647-1

**Published:** 2023-10-02

**Authors:** Louise Reveret, Manon Leclerc, Vincent Emond, Cyntia Tremblay, Andréanne Loiselle, Philippe Bourassa, David A. Bennett, Sébastien S. Hébert, Frédéric Calon

**Affiliations:** 1 Faculty of Pharmacy, Laval University, Quebec, QC Canada; 2 CHU de Quebec Research Center, 2705, Boulevard Laurier, Room T2-05, Québec, QC G1V 4G2 Canada; 3 Faculty of Medicine, Laval University, Quebec, QC Canada; 4 Rush Alzheimer’s Disease Center, Rush University Medical Center, Chicago, IL USA

**Keywords:** ACE2, Alzheimer’s disease, Cognitive dysfunction, Blood–brain barrier, Neuropathology

## Abstract

Cognitive decline due to Alzheimer’s disease (AD) is frequent in the geriatric population, which has been disproportionately affected by the COVID-19 pandemic. In this study, we investigated the levels of angiotensin-converting enzyme 2 (ACE2), a regulator of the renin-angiotensin system and the main entry receptor of SARS-CoV-2 in host cells, in *postmortem* parietal cortex samples from two independent AD cohorts, totalling 142 persons. Higher concentrations of ACE2 protein (p < 0.01) and mRNA (p < 0.01) were found in individuals with a neuropathological diagnosis of AD compared to age-matched healthy control subjects. Brain levels of soluble ACE2 were inversely associated with cognitive scores (p = 0.02) and markers of pericytes (PDGFRβ, p = 0.02 and ANPEP, p = 0.007), but positively correlated with concentrations of soluble amyloid-β peptides (Aβ) (p = 0.01) and insoluble phospho-tau (S396/404, p = 0.002). However, no significant differences in ACE2 were observed in the 3xTg-AD mouse model of tau and Aβ neuropathology. Results from immunofluorescence and Western blots showed that ACE2 protein is predominantly localized in microvessels in the mouse brain whereas it is more frequently found in neurons in the human brain. The present data suggest that higher levels of soluble ACE2 in the human brain may contribute to AD, but their role in CNS infection by SARS-CoV-2 remains unclear.

**Supplementary Information:**

The online version contains supplementary material available at 10.1186/s40478-023-01647-1.

## Introduction

Whether viral illnesses increase the risk of developing Alzheimer’s disease (AD) is a question raising considerable interest. In the light of the recent COVID-19 pandemics, an association between SARS-CoV-2 viral infection and cognitive decline due to AD or other causes has emerged [3, 24, 41, 59]. Risk factors for COVID-19 complications and fatalities are often the same as those for AD dementia—age, obesity, cardiovascular disease, hypertension, and diabetes mellitus [43, 47, 69, 86]. Notably, dementia per se is a strong predictor of COVID-19 mortality [55]. Using de-identified population-level electronic health records (EHR) from over 60 million individuals, a retrospective study showed that patients with dementia and COVID-19 had significantly worse outcomes (6-month hospitalization risk and mortality risk) than patients with dementia and no COVID-19 or patients with COVID-19 but no dementia [86]. In sum, people with dementia were disproportionately impacted by COVID-19, in terms of death and other clinical complications [17, 47, 68, 72, 92]. Whether this is due to age per se, a neuropathological AD diagnosis or other factors associated with cognitive decline is currently unknown.

Angiotensin-converting Enzyme 2 (ACE2) is a membrane carboxypeptidase that hydrolyzes Angiotensin I and Angiotensin II to respectively generate Angiotensin 1–9 and Angiotensin 1–7, which are part of the renin-angiotensin system (RAS) responsible for maintaining blood pressure, as well as fluid and salt balance [66]. ACE2 is highly expressed in the lung [18, 31] but also in other tissues such as the kidney, intestine, liver, testis and the brain [38, 48, 73]. The distribution of ACE2 in the brain is controversial, and earliest reports failed to identify the protein in the human CNS [22, 80]. Still, low levels of ACE2 mRNA were detected in the human brain using quantitative real**‐**time RT**‐**PCR [33]. Cerebral immunostaining was reported in endothelial and arterial smooth muscle cells [32], as well as in neurons [23]. More recently, single-cell RNA sequencing data have brought new insights on the cellular distribution of ACE2 transcripts in the brain vasculature. According to the Betsholtz mouse database, the expression of ACE2 is very high in microvascular mural cells (such as pericytes and venous vascular smooth muscle cells), but not in endothelial cells [35, 62, 84]. However, other databases report ACE2 mRNA expression in endothelial cells of mice [18, 94, 95]. So far, the available data suggest that the expression of ACE2 in the human brain is lower in both endothelial cells and pericytes compared to the mouse brain, albeit with important interregional variability [18, 34, 56, 90, 91].

ACE2 is also considered the main site of entry of SARS-CoV-2 into cells [40, 49, 70]. In previous outbreaks of SARS-CoV, which also use ACE2 as an entry point, the virus was detected in the brains of infected patients but reported almost exclusively in neurons [21, 32, 63, 78]. Indeed, CNS manifestations have been described in more than one third of hospitalized patients, especially those with severe conditions [17, 50, 54, 57, 67, 91, 92]. Thus, a higher ACE2 expression in the neurovascular unit of subjects with AD could provide additional entry points for SARS-CoV-2 into the CNS and facilitate neuroinfection.

First, to investigate whether ACE2 levels in the brain could be associated with cognitive dysfunction, we compared mRNA and protein levels of ACE2 in *postmortem* brain samples from individuals of two different cohorts, including subjects diagnosed with AD. In the first cohort from the Religious Order Study (n = 60), ACE2 protein levels were evaluated according to (i) the clinical diagnosis of no cognitive impairment (NCI), mild cognitive impairment (MCI), or AD; (ii) the neuropathological diagnosis of AD (ABC scoring) and the *antemortem* assessment of cognitive function. Associations between ACE2 and neurovascular markers were also examined. In the second cohort from other US sources (n = 82), brain levels of ACE2 protein and mRNA were investigated in individuals with a Braak-based neuropathological AD diagnosis. Finally, we compared the cellular localization of ACE2 between human and mouse brains and assessed ACE2 levels in a triple transgenic mouse model of AD neuropathology, the 3xTg-AD mouse.

## Materials and methods

### Human samples

#### Cohort #1

Gray matter samples from the Brodmann area 7 (BA7) corresponding to the posterior parietal cortex were obtained from participants in the Religious Orders Study *(Rush Alzheimer's Disease Center)*, an extensive longitudinal clinical and pathological study of aging and dementia [7, 8]. Each participant enrolled without known dementia and underwent annual structured clinical evaluations until death. A total of 21 cognitive performance tests were performed for each subject. At the time of death, a neurologist, blinded to all *postmortem* data, reviewed clinical data and rendered a summary diagnostic opinion regarding the clinical diagnosis proximate to death. Participants received a clinical diagnosis of no cognitive impairment (*n* = 20 NCI) or mild cognitive impairment (*n* = 20 MCI) or Alzheimer's disease (*n* = 20 AD), as previously described [7–9]. The neuropathological assessment for the subjects included in the present study was performed using the ABC scoring method found in the revised National Institute of Aging – Alzheimer’s Association (NIA‐AA) guidelines for the neuropathological diagnosis of AD [61]. Three different neuropathological parameters were evaluated for each subject: (A) the Thal score assessing phases of Aβ plaque accumulation [79], (B) the Braak score assessing neurofibrillary tangle (NFT) pathology [16] and (C) the CERAD score assessing neuritic plaque pathology [60]. These scores were then combined to obtain an ABC score, reported as "AX, BX, CX" with X ranging from 0 to 3 for each parameter [61]. Using the chart described in the revised NIA-AA guidelines [61], each ABC score was converted into one of four levels of AD neuropathological changes: not, low, intermediate or high. The maximal scores to be considered as low level are "A3, B1, C3" and "A1, B3 and C1", whereas the minimal score to qualify for the intermediate level is "A1, B2, C2". According to this chart, samples with intermediate or high pathological levels are consistent with a neuropathological diagnosis of AD because they have both extensive Aβ plaques and NFT, while those with no or low levels do not. Therefore, to establish a dichotomic classification in this study, individuals with intermediate or high levels of AD neuropathological changes were pooled in the AD group while participants with no or a low level of AD neuropathological changes were pooled in the Control group. Relevant data from the ROS samples used here and published previously [14, 15], are summarized in Table 1.

**Table 1:** Characteristics of Cohort #1 (Religious Order Study) and Cohort #2 (US other sources)

Religious Order Study (Cohort #1)			
Characteristics	Control	AD	Statistical Analysis
*N*	22	38	–
Men, %	41	29	C; Pearson test, x^2^ = 0.897; p = 0.3436
Mean age at death	86.7 (4.3)	87.5 (5.7)	Mann Whitney test, p = 0.5548
Post-mortem delay, hours	7.9 (5.1)	7.5 (5.1)	Mann Whitney test, p = 0.6648
Mean education, years	18.3 (3.5)	18.1 (3.1)	Mann Whitney test, p = 0.5236
Mean MMSE	25.0 (4.5)	21.6 (7.9)	Mann Whitney test, p = 0.0791
Global cognition score	− 0.32 (0.8)	− 0.94 (0.9)#	Mann Whitney test, p = 0.0044
apoE s4 allele carriage (%)	9	45 $	C; Pearson test, x^2^ = 8.182; p = 0.0042
Clinical diagnosis NCI/MCI/AD (n)	11/8/3	9/12/17	–
Thal amyloid score 0/1/2/3 (n)	7/13/2/0	0/3/15/20	–
Braak score 0/1/2/3 (n)	0/7/15/0	0/0/27/11	–
CERAD score 0/1/2/3 (n)	14/4/4/0	1/3/16/18	–
Parenchymal CAA stage in parietal cortex 0/1/2/3/4 (n)	15/4/1/1/0	18/7/5/2/3	C; Pearson test, x^2^ = 3.830; p = 0.4295
Presence of chronic cortical macroinfarcts 0/1 (n)	20/2	33/5	C; Pearson test, x^2^ = 0.224; p = 0.6363
Presence of chronic cortical microinfarcts 0/1 (n)	17/5	34/4	C; Pearson test, x^2^ = 1.627; p = 0.2021
Usage of antihypertensive medications 0/1 (n)	2/20	5/33	C; Pearson test, x^2^ = 0.224; p = 0.6363
Usage of diabetes medications 0/1 (n)	15/7	33/5	C; Pearson test, x^2^ = 3.032; p = 0.0816
Cerebellar pH	6.4 (0.37)	6.3 (0.36)	Mann Whitney test, p = 0.2933
Diffuse plaque counts in parietal cortex	3.8 (8.0)	20.3 (16.8)&	Mann Whitney test, p < 0.0001
Neuritic plaque counts in parietal cortex	1.3 (3.2)	15.7 (12.5)&	Mann Whitney test, p < 0.0001
Neurofibrillary Tangle Counts	0.09 (0.43)	2.92 (8.35)#	Mann Whitney test, p = 0.0096
Soluble Aβ40 concentrations, pmol/L	125.4 (245.9)	363.2 (695.2)1	Mann Whitney test, p = 0.0009
Soluble Aβ42 concentrations, pmol/L	299.6 (475.0)	1173.6 (503.9)&	Mann Whitney test, p < 0.0001
Soluble Aβ40/Aβ42 ratio	0.99 (1.09)	0.34 (0.59)	Mann Whitney test, p < 0.0001
Cyclophilin B in microvessel extracts (loading control) Claudin5 levels in microvessel extracts (normalized ROD) CD31 levels in microvessel extracts (normalized ROD)	2.74 (0.77) 1.17 (0.50) 0.45 (0.40)	2.66 (0.79) 1.16 (0.41) 0.41 (0.36)	Mann Whitney test, p = 0.7309 Mann Whitney test, p = 0.6613 Mann Whitney test, p = 0.7366

Other US sources (Cohort #2)			
Characteristics	Control	AD	Statistical Analysis
*N*	30	52	
Men, %	70	52	C; Pearson test, x^2^ = 2.561; p = 0.1095
Mean age at death	74.9 (9.7)	78.9 (14.0)	Unpaired t test, p = 0.0784
Post-mortem delay (h)	16.3 (4.9)	17.6 (4.8)	Unpaired t test, p = 0.2185
Braak stages 0–2/3–6 (n)	30/0	0/52	
Atherosclerosis (%)	n.d	61,5	

Cohort #1 characteristics (Religious Order Study): Participants were assigned to the “Control” or “AD” group based on the level of AD neuropathological changes associated with their ABC scores [61]. ABC scores were converted into one of the four levels of AD neuropathological changes (not, low, intermediate, or high) using the chart described in the revised NIA-AA guidelines [61]. Intermediate or high levels of AD neuropathological changes were assigned to the “AD” group, while those with no or a low level of AD neuropathological changes were rather assigned to the “Control” group [61]. Parenchymal CAA stages in parietal cortex were determined in the angular gyrus. Brain pH was measured in cerebellum extracts. Soluble Aβ peptide concentrations were determined by ELISA in whole homogenates of inferior parietal cortex. Values are expressed as means (SD) unless specified otherwise. Statistical analysis (compared to controls): Mann Whitney test: #p < 0.01; ¶p < 0.001; &p < 0.0001; Pearson test: £p < 0.01. Claudin5 and CD31 data in microvessel extracts were normalized with cyclophilin B as a loading control. Cohort #2 characteristics (Other US Sources): Brain samples of this cohort were provided by Harvard Brain Tissue Resource Center (Boston), Miller School of Medicine (Miami) and Human Brain and Spinal Fluid Resource Center (Los Angeles). Participants were assigned to the “Control” or “AD” group based on the Braak score. Values are expressed as means (SD). Statistical analysis (compared to controls): Unpaired t test, Pearson test. *AD* Alzheimer’s disease,* C* Contingency, *CAA* Cerebral amyloid angiopathy, *CERAD* Consortium to Establish a Registry for Alzheimer’s Disease, *MCI* Mild cognitive impairment, *MMSE* Mini-Mental State Examination, *NCI* Healthy controls with no cognitive impairment *ROD* Relative optical density

#### Cohort #2

Gray matter samples from the parietal cortex were obtained from 3 different institutions in the United States: 1- *Harvard Brain Tissue Resource Center,* Boston, Massachussetts, 2- *Brain Endowment Bank*, Miami, Florida. 3- *Human Brain and Spinal Fluid Resource Center*, Los Angeles, California [26]. All 82 parietal cortex samples were from the Brodmann area 39 (BA39), corresponding to the inferior region of the parietal cortex. Neuropathological diagnoses were based on Braak scores that were available for all cases. Because Thal and CERAD scores were not available for all cases, ABC diagnoses could not be used. To remain consistent with the ABC scoring used in Cohort #1, Braak scores of I or II were classified as Controls while Braak scores of III, IV, V and VI were considered as AD (Table 1). Frozen extracts from the parietal cortex were ground into a fine powder on dry ice with a mortar and pestle and kept at −80 °C.

### Protein fractionation from human parietal cortex homogenates (Cohort #1)

Each inferior parietal cortex sample (~ 100 mg) from Cohort #1 was sequentially sonicated and centrifuged to generate two protein fractions: a Tris-buffered saline (TBS)‐soluble fraction containing soluble intracellular, nuclear and extracellular proteins and a detergent‐soluble protein fraction containing membrane-bound proteins extracted with a mix of detergents (0.5% Sodium dodecyl sulfate (SDS), 0.5% deoxycholate, 1% Triton), as previously reported [81, 82]. Protein contents of supernatants were quantified using a bicinchoninic acid assay (Thermofisher cat: P123227). Protein homogenates in Laemmli were prepared as described below.

### Isolation of human brain microvessels (Cohort #1)

The method used to generate microvessel-enriched extracts from frozen human parietal cortex samples has been described in our previous publications [13–15]. Briefly, this method consists of a series of centrifugation steps, including one density gradient centrifugation with dextran, after which the tissue is filtered through a 20-µm nylon filter. This generates two fractions: the material retained on the filter consists in cerebral microvessels (isolated microvessel-enriched fraction), whereas the filtrate consists in microvessel-depleted parenchymal cell populations. These fractions were homogenized in lysis buffer (150 mM NaCl, 10 mM NaH_2_PO_4_, 1% Triton X-100, 0.5% SDS, and 0.5% sodium deoxycholate), so they contain all proteins (intracellular and membrane-bound) and are used in immunoblotting.

Alternatively, isolated microvessels on the filter were resuspended in 3 ml of microvessel isolation buffer (HBSS; 15 mM HEPES, 147 mM NaCl, 4 mM KCl, 3 mM CaCl_2_, and 12 mM MgCl_2_) with 1% BSA and protease and phosphatase inhibitors and spun at 2000 g for 10 min at 4 °C. The supernatant was discarded and the pellet was resuspended in 100 μl of phosphate buffer saline (PBS). These cerebrovascular extracts were then deposited on glass slides (5 μl per slide) and left at RT for 30 min to allow adhesion. Afterwards, they were fixed using a 4% paraformaldehyde solution in PBS for 20 min at RT and processed for immunostaining.

Cerebral fractions enriched and depleted in endothelial cells were evaluated using immunoblotting of vascular and neuronal markers, as shown previously [14]. Isolation of brain microvessels was performed on human samples of Cohort #1 (final n = 56) and the fractions were used for Western blot analysis and immunostaining.

### Protein and RNA extraction and RT-qPCR analysis (Cohort #2)

Approximatively 50 mg of this fine powder were used for total protein extraction. Then, a lysis buffer (50 mM Tris–HCL to pH 7.4, 150 mM NaCl, 1% triton and 0.5% sodium deoxycholate) containing protease (complete 25 X and Pepstatin A) and phosphatase inhibitors (sodium fluoride and sodium vanadate) was added. The sample solution was homogenized on ice by sonication using a Sonic Dismembrator (Fisher, Pittsburgh, PA) with two 10-s pulses and a 30-s stop between steps. Samples were centrifuged 20 min at 10,000 g at 4 °C. Protein contents of supernatants were quantified using a bicinchoninic acid assay (Thermofisher cat: P123227). Protein homogenates in Laemmli were prepared as described below.

Approximately 100 mg of powderized parietal cortex were used for total RNA extraction with TRIzol (Ambion) using manufacturer's instructions. The resulting RNA pellet was resuspended in 80 μL RNAse-free water and incubated 10 min at 57 °C. The RNA concentration was measured with an Infinite F200 (Tecan). The reverse transcription (RT) was performed with 1 µg of RNA. As a first step, genomic DNA was removed following the AccuRT Genomic DNA removal protocol (Applied Biological Materials, ABM, Vancouver, Canada). Then the RT master mix (ABM) was added to RNA samples and incubated (10 min at 25 °C, 50 min at 42 °C and 5 min at 85 °C) as per the manufacturer’s protocol. All qPCR experiments were performed on the LightCycler 480 (Roche) with the BrightGreen mix (ABM) and primers at 10 µM. After enzyme activation for 10 min at 95 °C, 50 cycles were performed (15 s at 95 °C and 1 min at 60 °C), followed by 1 s at 95 °C and 1 min at 45 °C. Reference gene GAPDH (primers Forward: TCTCCTCTGACTTCAACAGCGAC and Reverse:CCCTGTTGCTGTAGCCAAATTC) was used to normalize the mRNA expression. The relative amounts of each transcript were calculated using the comparative Ct (2-ΔΔCt) method. For *Ace2* qPCR (primers Forward: GTGCACAAAGGTGACAATGG and Reverse: GGCTGCAGAAAGTGACATGA), 12 controls and 19 AD individuals were used. We used a cut-off of ≥ 35 cycles for these samples.

### Isolation of murine brain microvessels and protein fractionation

All experiments were performed in accordance with the Canadian Council on Animal Care and were approved by the Institutional Committee at the Centre Hospitalier de l’Université Laval (CHUL). Four (4) or six (6)-, 12- and 18-month-old 3xTg-AD (APPswe, PS1M146V, tauP301L) mice produced at our animal facility were used in equal numbers of males and females in each group. These mice show progressive accumulation of Aβ plaques and neurofibrillary tangles, which are detectable at 12 months and are widespread after 18 months [12, 19]. Mice were fed a standard chow (Teklad 2018, Harlan Laboratories, Canada) from breeding to 5 months of age. Mice were then fed a control diet (CD; 20%kcal from fat) or a high-fat diet (HFD; 60%kcal from fat) from 6 to 18 months of age, in order to worsen neuropathology and memory performance and to induce metabolic impairments [5, 44, 75, 83], which are also associated with a higher risk of developing severe SARS-CoV-2 infections [1, 43]. As previously described [77], mice were killed under deep anesthesia (100 mg/kg ketamine, 10 mg/kg xylazine) via terminal intracardiac perfusion of PBS containing protease and phosphatase inhibitors. The brains were immediately collected and transferred into ice-cold perfusion buffer, where meninges, cerebellum and brainstem were removed. The parieto-temporal cortex was rapidly dissected and frozen at − 80 °C until processed for protein extraction. TBS-soluble (intracellular and extracellular fraction) and detergent-soluble fractions (membrane fraction) were prepared as described above. Alternatively, brain tissue was chopped and frozen in 0.5 mL of HBSS containing 0.32 M sucrose until processed for microvessel enrichment, which was performed as described above for human samples. Fractions enriched and depleted in cerebrovascular cells were either homogenized in lysis buffer (total proteins including intracellular and membrane-bound) for Western blot analysis or deposited on glass slides for immunofluorescence, as above.

### Western blot analysis

Microvessel protein homogenates from human parietal cortex and murine whole brain extracts were added to Laemmli’s loading buffer and heated 10 min at 70 °C. TBS- and detergent-soluble fractions from homogenates of human parietal cortex were also added to Laemmli’s loading buffer and heated 5 min at 95 °C. Equal amounts of proteins per sample (8 µg for both human and murine brain microvessel extracts and 12 µg for protein homogenates of human parietal cortex, 15 µg for protein homogenates of mouse brain) were resolved by sodium dodecyl sulphate–polyacrylamide gel electrophoresis (SDS-PAGE). All samples, loaded in a random order, were run on the same immunoblot experiment for quantification. Proteins were electroblotted on PVDF membranes, which were then blocked during 1 h with a PBS solution containing 5% non-fat dry milk, 0.5% BSA and 0.1% Tween-20. Membranes were then incubated overnight at 4 °C with primary antibodies (rabbit anti-ACE2, #ab108252, 1:1000, rabbit anti-TMPRSS2 #ab109131, 1:1000). Membranes were then washed three times with PBS containing 0.1% Tween-20 and incubated during 1 h at room temperature with the secondary antibody (goat/donkey anti-rabbit HRP Jackson ImmunoResearch Laboratories, West Grove, PA; 1:60,000 or 1:10,000 in PBS containing 0.1% Tween-20 and 1% BSA). Densitometric analysis was performed using ImageLab (Bio-Rad). Uncropped gels of human samples immunoblot assays are shown in the Additional file 2: Material (Additional file 1: Figs. S6 and S7).

### Immunostaining

To demonstrate ACE2 localization in *postmortem* human brain tissue, we tested a number of commercially available antibodies using a wide range of immunostaining protocols. Immunostaining was performed on formalin-fixed, paraffin-embedded (FFPE) tissue Sects. (6 µm) of human parietal cortex, on fresh-frozen (FF) tissue Sects. (12 µm) from human and mouse hippocampus, as well as on human and murine isolated brain microvessels (see below). Briefly, FFPE sections were deparaffinized in CitriSolv hybrid and rehydrated with decreasing concentrations of ethanol in water. Antigen retrieval was then performed by boiling slides in Tris buffer (10 mM, pH 9.0) with 1 mM EDTA and 0.05% (v/v) Tween-20 in a microwave for 15 min and letting them cool for 30 min at room temperature. Sections were quenched with 50 mM NH_4_Cl, digested with trypsin 0.1% (w/v, Sigma-Aldrich) at 37 °C for 15 min, and incubated in Tris-buffered saline (TBS) with 0.3 M glycine for 15 min. Sections were then blocked sequentially with Bloxall, avidin/biotin blocking kit (Vector Laboratories, CA) and Superblock (Thermo) with 0.2% Triton-X100, and used for immunohistochemistry. FF sections and brain microvascular fractions were kept at − 80 °C until use, then vacuum-dried at 4 °C and fixed in 4% (w/v) paraformaldehyde (pH 7.4) for 20 min at room temperature. All sections were then blocked and permeabilized for 1 h with Superblock containing 0.2% (v/v) Triton X-100. Incubation with primary antibodies (various rabbit anti-ACE2, mouse anti-NeuN MAB377, goat anti-collagen IV AB789) was performed overnight at 4 °C in Superblock with 0.05% Tween-20. For immunohistochemistry, after washing in PBS, sections were incubated with biotinylated secondary antibodies (Jackson Immunoresearch) and then with streptavidin-HRP (ABC Elite kit). ACE2 localization was revealed using the ImmPACT AMEC red substrate and nuclei were counterstained with Mayer’s hematoxylin. For immunofluorescence, after washes, secondary antibodies (conjugated to Alexa Fluor 555, 647 and 750, which use channels with less autofluorescence) were added to sections for 1 h. Slides were then sequentially incubated with 4′,6-diamidino-2-phenylindole (DAPI) and TrueBlack Plus (Biotium, CA) to quench lipofuscin autofluorescence. Photomicrographs were recorded with a Cytation 5 or EVOS fl Auto Imaging System (Thermo Fisher).

### Data and statistical analysis

An unpaired Student’s t-test was performed when only two groups were compared, with a Welch correction when variances were not equal. If the data distribution of either one or both groups failed to pass the normality tests (Shapiro–Wilk test or Kolmogorov–Smirov test), groups were compared using a non-parametric Mann–Whitney test. When more than two groups were compared, parametric one-way ANOVA followed by Tukey’s multiple comparison tests or two-way ANOVA were used. If criteria for variance (Bartlett’s) or normality were not met, non-parametric Kruskal–Wallis ANOVA followed by Dunn’s multiple comparison tests were used. If needed, data were log transformed to normalize distributions. For all data, statistical significance was set at P < 0.05. Individual data were excluded for technical reasons or if determined as an outlier using the ROUT (1%) method in GraphPad Prism. Linear correlation analysis was used to determine correlation coefficients between ACE2 and *antemortem* evaluation, neuropathological markers or BBB markers. All statistical analyses were performed with Prism 9 (GraphPad, San Diego, CA, USA) or JMP (version 16; SAS Institute Inc., Cary, IL) software.

## Results

### Association between ACE2 in the parietal cortex, the neuropathological diagnosis of AD and cognitive scores

**Fig. 1:**
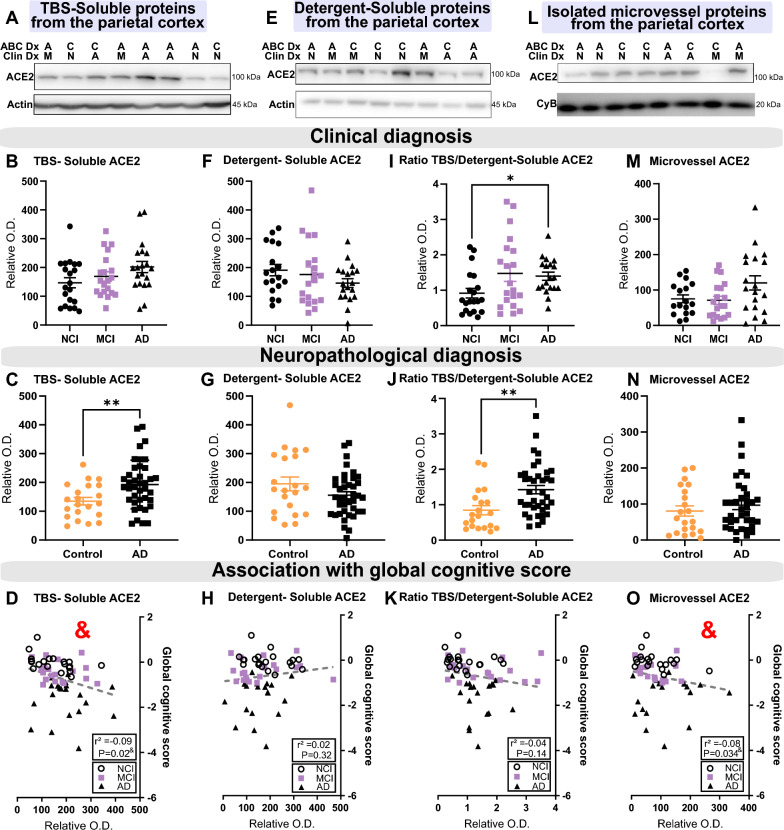
Levels of TBS-Soluble ACE2 protein are higher in AD individuals and are negatively correlated with global cognitive score*.* Parietal cortex levels of ACE2 protein from Cohort #1 were determined by Western blotting after SDS-PAGE (10% acrylamide) of three fractions: a TBS-soluble fraction **A**–**D** a detergent-soluble fraction **E**–**H** and a microvessel-enriched fraction **L–O**. No statistical difference was detected for ACE2 in the three fractions when subjects were classified according to *antemortem* clinical diagnosis (**B**, **F**, **M**). However, the TBS/Detergent-Soluble ACE2 ratio was higher in clinical AD subjects **I**. Levels of the ACE2 protein were higher in the TBS-soluble fraction and in the ratio TBS/Detergent-Soluble in individuals with a neuropathological diagnosis of AD based on ABC scoring **C**, **G**, **J, N**. TBS-soluble ACE2 and microvascular ACE2 levels were negatively correlated with the global cognitive score **D**, **O**. An equal amount (12 µg) of proteins per sample for both TBS-soluble and detergent soluble fractions was loaded and 8 µg of proteins per sample was loaded for microvessel-enriched fractions. All samples, loaded in a random order, were run on the same gel and transferred on the same membrane before immunoblotting for quantification. Examples were taken from the same experiment, and consecutive bands loaded in random order are shown. Actin and cyclophilin B are shown as loading controls. Data are represented as a scatterplot. A very high outlier has been removed in the clinical AD group in I, J and K. Horizontal lines indicate mean ± SEM. Statistical analysis: Two groups: Mann–Whitney test **p < 0.01; Three groups: Kruskal–Wallis followed by a Dunn’s multiple comparisons *p < 0.05, Coefficient of determination & p < 0.05. *ACE2* Angiotensin-Converting Enzyme 2*, A or AD* Alzheimer’s disease,* C* Control, *Clin Dx* Clinical diagnosis, *ABC Dx* ABC neuropathological diagnosis, *CyB* Cyclophilin B*, M or MCI* Mild cognitive impairment*,* N or NCI Healthy controls with no cognitive impairment, *O.D.* optical density, SEM Standard error of the mean*, TBS* Tris-Buffered Saline

**Fig. 2:**
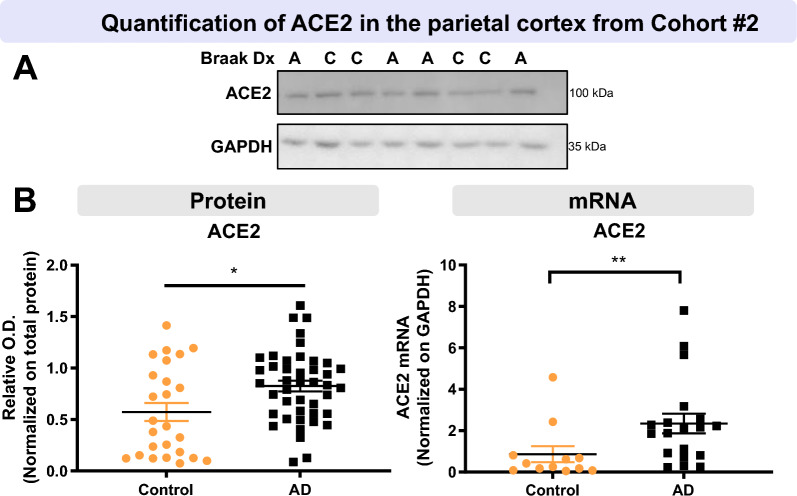
Higher ACE2 protein and mRNA levels in parietal cortex of AD participants from the second cohort. AD subjects from Cohort #2 had higher levels of ACE2 protein and mRNA compared to controls. Diagnosis was determined using Braak staging. ACE2 levels were determined by Western blotting after SDS-PAGE (10% acrylamide) and qPCR analysis **A**, **B**. Statistical analysis: Mann–Whitney test *p < 0.05, Unpaired t-test **p < 0.01. All samples, loaded in a random order, were run on the same immunoblot experiment for quantification. Examples were taken from the same immunoblot experiment, and consecutive bands loaded in random order are shown. GAPDH is shown as a loading control. Data are represented as a scatterplot. Horizontal lines indicate mean ± SEM. *A* Alzheimer’s disease (Braak scores III-VI), ACE2 Angiotensin-Converting Enzyme 2*, C* Control (Braak scores I or II), *Dx* Diagnostic, *GAPDH* Glyceraldehyde-3-phosphate dehydrogenase*, O.D.* Optical Density, *SEM* standard error of the mean

Table 1 summarizes the clinical and biochemical data from both cohorts, showing that subjects with AD displayed higher tau and Aβ pathologies but comparable levels of endothelial proteins cyclophilin B, claudin-5 and CD31. ACE2 protein levels weremeasured in TBS-soluble (cytosolic, extracellular, nuclear and secreted proteins), detergent-soluble (membrane-bound proteins) and microvessel-enriched fractions (vascular proteins) from parietal cortex samples. Representative Western immunoblots of ACE2 and analyses are shown in Fig. 1 for Cohort #1 and Fig. 2 for Cohort #2. A band migrating at approximately 100 kDa, corresponding to full-length ACE2, was observed in each fraction (Figs. 1, 2).

We first evaluated ACE2 protein levels in extracts from the parietal cortex of 60 individuals from Cohort #1, ROS. When the subjects were classified according to the neuropathological ABC diagnosis, higher levels of ACE2 protein were found in TBS-soluble fractions from AD subjects compared to non-AD participants (p = 0.0087) (Fig. 1C). This rise in soluble ACE2 was more prominent in AD ApoE4 carriers (Additional file 1: Fig. S2). When the subjects were classified according to the clinical diagnosis, only a non-significant trend towards higher ACE2 concentrations was observed in the TBS-soluble fraction, using a non-parametric Kruskal–Wallis ANOVA (p = 0.1471) (Fig. 1B). However, the difference between AD and Controls/NCI was statistically significant when this comparison was performed only in individuals with parenchymal cerebral amyloid angiopathy (p = 0.0022) (pCAA; Additional file 1: Fig. S1A, D). On the other hand, ACE2 levels assessed in the detergent-soluble fraction, enriched for membrane-associated proteins, remained similar between groups (Fig. 1E–G). However, the ratio of the TBS/Detergent-soluble forms was significantly higher in patients diagnosed with AD clinically and neuropathologically (p = 0.0272 and p = 0.0016, respectively) (Fig. 1I–J).

We next measured ACE2 protein levels in microvessel extracts. Given the high interindividual variability, only a non-significant trend towards higher ACE2 protein levels was observed in individuals with an AD clinical diagnosis (p = 0.1712) (Fig. 1L–N). However, the difference with Controls became significant when including only AD patients with pCAA (Additional file 1: Fig. S1C, F).

**Fig. 3:**
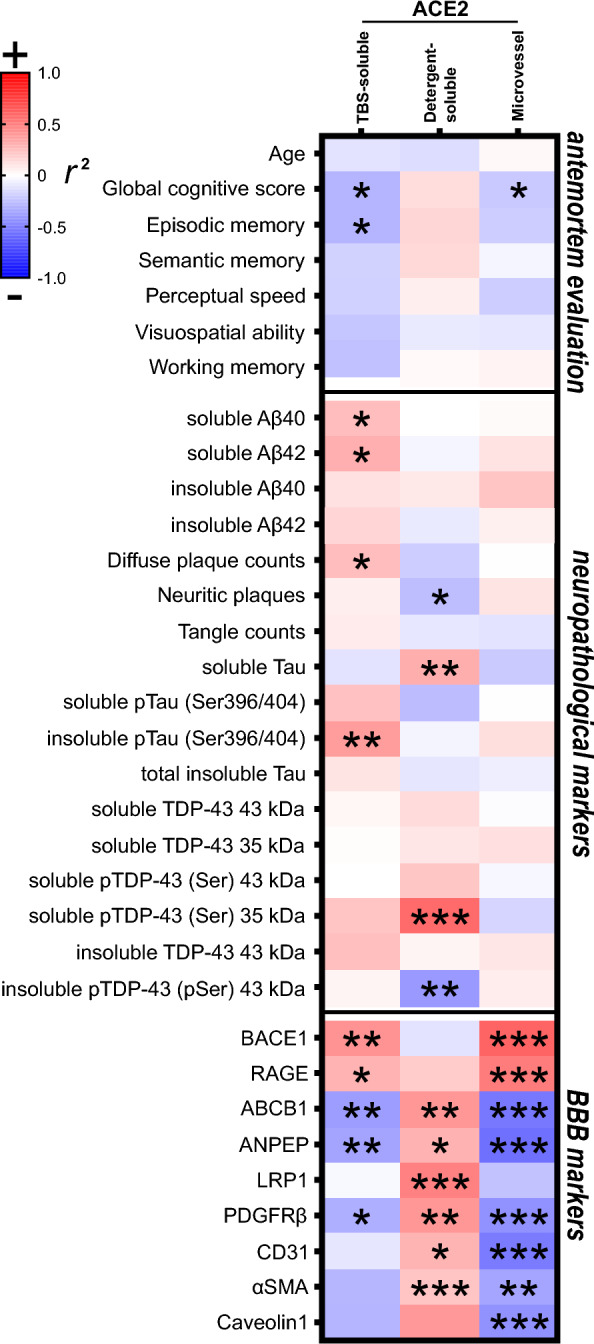
ACE2 protein in TBS-soluble and microvascular protein fractions show opposite relationships with detergent-soluble ACE2 when correlating with AD markers. Levels of ACE2 were investigated for their associations with several AD-relevant variables in human parietal cortex. Soluble and insoluble proteins were quantified in TBS-soluble and detergent-insoluble (formic acid) fractions, respectively, whereas BBB markers were measured in microvascular fractions [14, 15, 81, 82]. Linear regressions were performed to generate coefficients of determination (*r*^*2*^). Heat-map of hierarchical clustering analysis of correlation coefficients shows partial correlation analyses with *antemortem* evaluation, neuropathological markers and BBB markers. The significance of the correlation (inverse is highlighted in blue, positive in red) between two elements is entered in the associated box. Graph examples are shown in Figure S4. Statistical analysis: Coefficient of determination *p < 0.05, **p < 0.01 and ***p < 0.001. All proteins presented in these heat-maps were determined by western blot analysis except Aβ peptides, which were quantified using ELISA. Total soluble and insoluble Tau was detected using Tau (640–680) antibody. AD2 antibody binds to Tau phosphorylated at S396. Phosphorylated TDP43 antibody binds pSer409/410. *ABCB1* ATP Binding Cassette Subfamily B Member 1*, ACE2* Angiotensin-Converting Enzyme 2 *ANPEP* Aminopeptidase N, *BBB* Blood–brain barrier, *BACE1* Beta-Secretase 1*, CD31 or PECAM1* Platelet endothelial cell adhesion molecule, *LRP1* Low density lipoprotein receptor-related protein 1*, PDGFRβ* Platelet Derived Growth Factor Receptor Beta *RAGE* Receptor for Advanced Glycation Endproducts, *αSMA* alpha smooth muscle actin, *TDP-43* TAR DNA binding protein 43, *TBS* Tris-Buffered Saline

Interestingly, the levels of ACE2 found in TBS-soluble and microvessel-enriched fractions were inversely associated with *antemortem* global cognitive scores (Fig. 1D, O and Fig. 3). This association remained significant after adjustment for age at death and sex.

To corroborate these results, we performed Western immunoblots on a second series of human brain samples from Cohort #2 [26] (Fig. 2A). Consistently, higher levels of ACE2 protein were detected in individuals with a Braak-based diagnosis of AD (Fig. 2B), in association with higher *Ace2* mRNA levels (Fig. 2B), suggesting a regulation at the transcriptional level. No difference was observed in the levels of transmembrane protease serine type 2 (TMPRSS2) (Additional file 1: Fig. S3), a protein that plays a key role in SARS-CoV-2 infection by activating the spike protein, facilitating entry into target cells using ACE2 [40].

### TBS-soluble ACE2 is positively associated with clinical, neuropathological, and vascular markers of AD, while detergent-soluble ACE2 displays opposite trends

Hierarchical clustering of correlation coefficients (strength of association) was performed to identify variables associated with differences in ACE2 in Cohort #1. Although the leading risk factor for AD is age, no significant correlation was found between ACE2 levels in all fractions tested and the ages of death (Fig. 3, Additional file 1: Fig. S4), which were equivalent between groups (Table 1). These observations suggest that the greater soluble ACE2 in individuals with AD in the ROS cohort was not driven by age. However, the age interval (74–98 years) was too small to detect an effect of aging per se on cerebral ACE2. Beside the inverse association with global *antemortem* cognitive scores of participants (*r*^2^ = -0.09, P < 0.05, Figs. 1D and 3), higher *postmortem* TBS-soluble concentrations of ACE2 were also significantly correlated with failing episodic memory, a domain predominantly affected in AD (Fig. 3).

Associations were then examined with neuropathological markers of AD, previously assessed in the parietal cortex from the same sample series (Fig. 3). Levels of TBS-soluble ACE2 were positively associated with AD markers like diffuse plaque counts, soluble Aβ levels and insoluble phospho-tau (pS396/404 epitope) (Fig. 3). In contrast, levels of detergent-soluble ACE2 were negatively associated with the insoluble phosphorylated form of TAR DNA-binding protein 43 (TDP-43) (which is higher in AD [15]) but positively with soluble phospho-TDP-43 C-terminal fragment migrating at approximately ~ 35 kDa (which is lower in AD [15]) and soluble tau (Fig. 3). Similarly, neurovascular proteins such as platelet-derived growth factor receptor β (PDGRFRβ), and ABCB1 correlated positively with membrane-bound ACE2 but inversely with TBS-soluble and vascular forms of ACE2, which in turn correlate with β-secretase 1 (BACE1) and advanced glycosylation end product-specific receptor (RAGE), proteins involved in the formation and accumulation of Aβ (Fig. 3).

Single-cell RNA sequencing data in the mouse and human brain show that ACE2 mRNA expression is enriched in pericytes [34, 62], and PDGFRβ, a marker of mural cells including pericytes, is reduced in AD [15, 58]. Here, we found that microvascular PDGFRβ and aminopeptidase N (ANPEP) levels were negatively correlated with TBS-soluble ACE2 levels but were positively associated with detergent-soluble ACE2 levels (Fig. 3, Additional file 1: Fig. S4), suggesting a possible release of ACE2 from membranes linked with pericyte-related dysfunctions at the blood–brain barrier (BBB).

Together, these results suggest that the elevation of ACE2 in TBS-soluble and, to a lesser extent, in microvessel fractions are associated with more advanced Aβ and tau pathologies and with a pattern of changes in vascular proteins consistent with AD progression. By contrast, membrane-bound ACE2 exhibited opposite trends and was strongly associated with reduced TDP-43 proteinopathy and consolidated BBB markers.

### ACE2 is observed in human and murine neurons and cerebral vessels

**Fig. 4:**
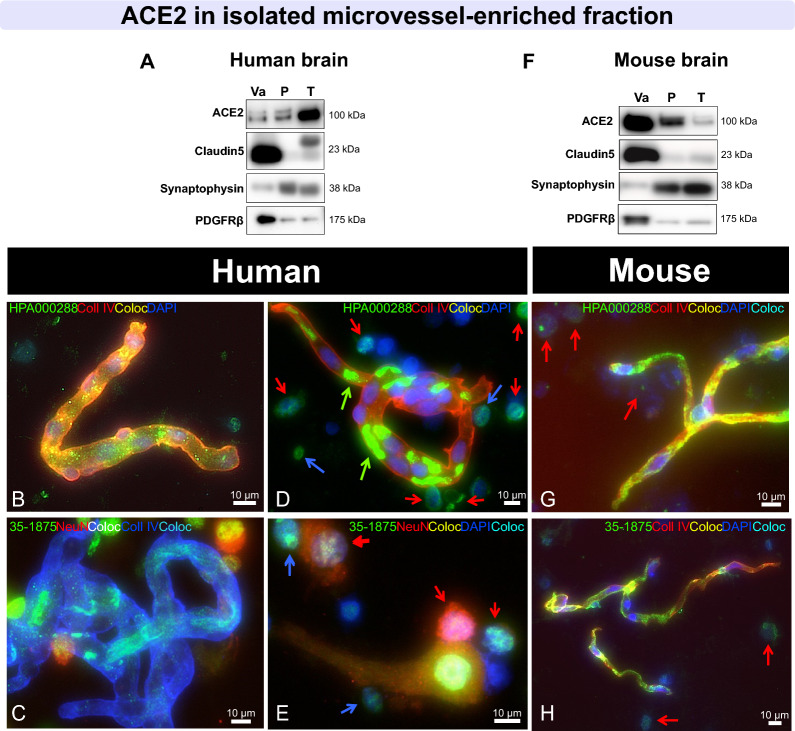
ACE2 immunosignal is strong in mouse microvessels, but also found in neurons in human brain extracts. **A**, **F** Immunoblotting detection of ACE2 in human (parietal cortex) **A** and mouse (whole brain) **F** vascular fractions “*Va*”, compared to postvascular parenchymal samples depleted in vascular cells “*P*” and total homogenates “*T*”. SDS-PAGE was performed on a 4–20% acrylamide gradient gel. For comparison purposes, synaptophysin (synaptic/neuronal marker), Claudin5 (endothelial marker), and PDGFRβ (a marker of mural cells, including pericytes), are also shown. In the mouse brain, ACE2 is highly enriched in microvessels compared to the postvascular fraction, different to what is observed in the human **A**, **F**. **B**–**E**, **G**, **H** Representative immunostaining of ACE2 (green) in human **B**–**E** and murine cerebrovascular fractions **G**, **H**, with collagen IV (endothelial marker) in red **B**, **D**, **G**, **H** or blue **C**, **E**, as well as NeuN (neuronal marker) in red **C**, **E** and DAPI (nuclei) in blue **B**–**H**. In human samples, ACE2 staining is most frequently observed in neurons, whereas ACE2 staining is concentrated in murine microvessels. Red arrows point to ACE2 + /NeuN + cells, blue arrows to ACE2 + /NeuN- cells, and green arrows to erythrocytes. ACE2 antibodies: rb mAb #ab108252 **A**, **F**, rb pAb #HPA000288 **B**, **D**, **G** and #35–1875 **C**, **E**, **H**. Scale bar: 10 µm. *ACE2* Angiotensin-Converting Enzyme 2, *Coll IV* Collagen IV *Coloc* Colocalization, *NeuN* Neuronal nuclear protein, *PDGFRβ* Platelet Derived Growth Factor Receptor Beta*. mAb* Monoclonal antibody, *pAb* Polyclonal antibody

Localizing ACE2 within the neurovascular unit at the interface between the blood and the brain can provide basic information about SARS-CoV-2 penetration into the CNS. Therefore, we sought to determine whether ACE2 protein was enriched in brain microvessel extracts compared to post-vascular parenchymal fractions and unfractionated homogenates from human (parietal cortex) and mouse (whole brain) samples (Fig. 4). We found a strong enrichment of ACE2 in murine cerebral microvessels, along with endothelial marker Claudin5 and a marker of mural cells including pericytes, PDGFRβ (Fig. 4F). However, in human brain samples, ACE2 protein levels were more comparable between microvessel and parenchymal fractions, the latter being enriched in the neuronal marker synaptophysin (Fig. 4A). Thus, these Western blot results suggest that the localization of ACE2 in the brain differs between both species, with a cerebrovascular predominance in the mouse that was not observed in humans.

**Fig. 5:**
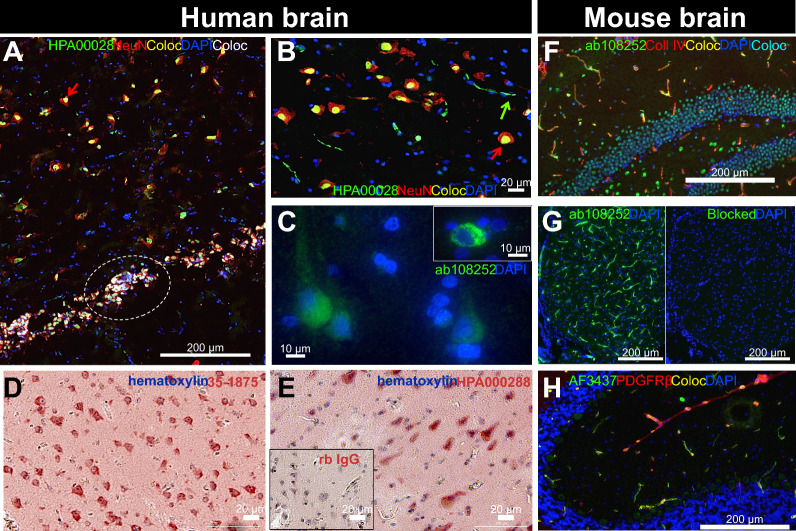
In tissue sections, ACE2 immunostaining is predominantly observed in neurons in the human brain and in the cerebrovasculature in mice. **A**–**E** Representative immunostaining of ACE2 (green in IF or red in IHC) in fresh frozen human hippocampus **A**, **B**, formalin-fixed paraffin-embedded parietal cortex **C**–**E**, and in murine fresh frozen hippocampal **F** and cerebellar **G**–**H** sections. Inlaid in **G**, the primary mAb was preblocked with its immunogenic peptide, preventing epitope binding. For immunofluorescence, NeuN (neuronal marker) **A**, **B**, collagen IV (endothelial marker) **F** or PDGFRβ (a marker of mural cells including pericytes) **H** are in red, and DAPI (nuclei) is in blue **A–C**, **F–H**. For immunohistochemistry, sections were counterstained with hematoxylin (nuclei) **D**, **E**, and negative control is inserted in **E**. In human samples, strong ACE2 staining is observed in large and small neurons, whereas in mice, neuronal signal is moderate and vascular ACE2 staining is strong and colocalizes well with PDGFRβ. Red arrows point to ACE2 + /NeuN + cells and green arrows to erythrocytes; dashed white line highlights small ACE2 + /NeuN + cells that stain strongly for DAPI. ACE2 antibodies: rabbit mAb #ab108252 **C**, **G**, rabbit pAb #HPA000288 **A**, **B, E**, **F** and #35–1875 **D** or goat pAb #AF3437. Scale bars: 200 µm **A**, **F**, **G**, **H**, 20 µm **B**, **D**, **E** and 10 µm **C**. *ACE2* Angiotensin-Converting Enzyme 2, *Coll IV* Collagen IV, *Coloc* Colocalization, *NeuN* Neuronal nuclear protein, *PDGFRβ* Platelet Derived Growth Factor Receptor Beta *mAb* monoclonal antibody, *pAb* polyclonal antibody

To confirm the cellular localization of ACE2, immunostaining was also performed on cerebrovascular extracts (Fig. 4B-E, G, H). A moderate immunofluorescent signal was detected inside microvessels isolated from human brains (collagen IV-positive, Fig. 4B, C) and NeuN-positive neurons (Fig. 4C, E). By contrast in the mouse, ACE2 immunosignal was intense in microvessels and colocalized well with collagen IV (Fig. 4G, H). To validate immunostaining in human tissue sections, nine anti-ACE2 antibodies were used in human testis samples where ACE2 is highly expressed in Leydig and Sertoli cells (Additional file 1: Fig. S5). All antibodies showed a clear signal in this tissue. However, detection of ACE2 in the human brain, where levels are at least 20 times lower (results not shown), was challenging with a majority of antibodies, and only antibodies ab108252, HPA000288 and 35–1875 gave a satisfactory signal. In human hippocampal sections, ACE2 was detected in NeuN-positive neurons, particularly in large ones staining weakly for DAPI and in small ones with a strong DAPI signal (Fig. 5A, B). In sections of human parietal cortex, ACE2 detection was also more prominent in neuron-like cells (Fig. 5C, D, E). On the other hand, in mouse sections, ACE2 staining was more intense in the cerebrovasculature and colocalized neatly with PDGFRβ, indicating an expression in mouse pericytes (Fig. 5F–H). Negative controls and channel splits are shown in Additional file 1: Fig. S6. These results are consistent with Western blot data, showing that ACE2 can be detected in neuron-like cells in the human brain, whereas it is concentrated in cerebrovascular cells in the mouse.

### Microvascular and whole-brain ACE2 protein levels are not altered in a mouse model of AD

**Fig. 6:**
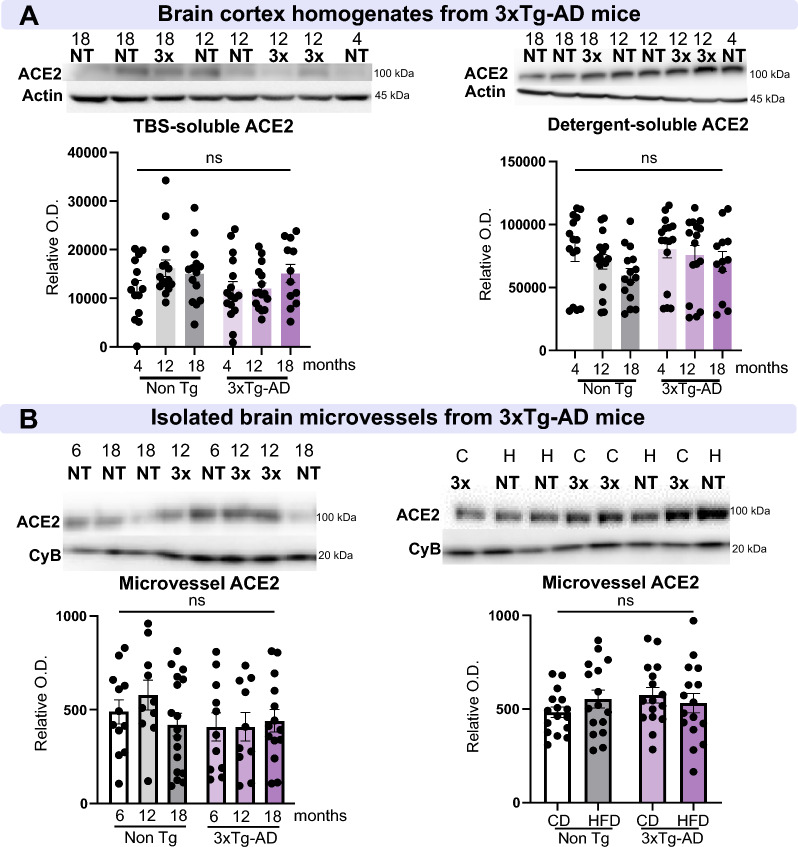
ACE2 levels are not altered in a model of AD, the 3xTg-AD mouse. **A** Determination of ACE2 levels by Western immunoblotting in brain homogenates from NonTg and 3xTg-AD mice aged 4, 12, and 18 months. No difference was observed in TBS-soluble and detergent-soluble ACE2. **B** In brain microvessel-enriched fractions from NonTg and 3xTg-AD mice aged 6, 12, 18 months, and in 18-month-old animals fed either a control diet or a high fat diet, no difference was observed in microvascular ACE2 levels. ACE2 levels in the mouse brain are not influenced by age or a diet that exacerbates AD-like neuropathology. Examples were taken from the same immunoblot experiment, and consecutive bands loaded in random order are shown. Actin and cyclophilin B are shown as loading controls. Data are represented as mean ± SEM. Statistical analysis: Kruskal–Wallis, ns, non-significant. *ACE2* Angiotensin-Converting Enzyme 2, *AD* Alzheimer’s disease, *Non-Tg,/NT* Non-transgenic mice, *3* × 3xTg-AD mice, *CD/C* Control diet*, CyB* cyclophilin B, *HFD/H* high-fat diet, *O.D.* optical density, *SEM* Standard error of the mean, *TBS* Tris-Buffered Saline

To probe whether changes in ACE2 could be a consequence of classical tau and Aβ neuropathology, we used the triple transgenic mouse model of AD (3xTg-AD) [64], which develops Aβ plaques and neurofibrillary tangles (NFT) by 12 months of age. We quantified ACE2 protein levels in both 3xTg-AD and non-transgenic mice from two different cohorts: (i) mice of 4 or 6, 12, and 18 months of age, (ii) and 18-month-old mice fed either a control or a HFD that exacerbates neuropathology [83] (Fig. 6). No significant change was observed in protein levels of ACE2 in TBS-soluble or detergent-soluble fractions according to genotype and age (Fig. 6A). Similarly, ACE2 in cerebrovascular fractions did not vary according to genotype, age, and diet (Fig. 6B). These results suggest that the development of human tau and Aβ neuropathology in mice is insufficient to increase murine ACE2 levels, even when combined with aging and HFD, two risk factors for both AD and COVID-19 infection.

## Discussion

This present *postmortem* study investigated ACE2 concentrations in the brain of individuals with AD from two different cohorts. We assessed ACE2 protein levels in all subjects and mRNA expression in a subset. We observed a significant relationship between ACE2 levels, the neuropathological diagnosis of AD, and *antemortem* cognitive evaluation. Overall, our data indicate that (1) levels of TBS-soluble ACE2 in the parietal cortex were higher in persons with AD when compared to control subjects, accompanied by an elevation in ACE2 mRNA transcripts; (2) lower cognitive scores were associated with higher levels of ACE2 in TBS-soluble and cerebrovascular fractions; (3) an apparent transfer of ACE2 from membranes to a soluble compartment was associated with pericyte loss and other markers of AD progression; (4) ACE2 levels remained unchanged in an animal model of AD-like neuropathology; (5) whereas ACE2 was highly concentrated in microvessels in the mouse brain, it was more frequently found in neurons in the human brain. Such a series of observations highlight that an AD diagnosis is associated with higher levels of specific forms of ACE2 in the brain, which might contribute to the higher risk of SARS-CoV-2 CNS infection in cognitively impaired individuals.

### Higher levels of soluble ACE2 are associated with AD and cognitive decline

The present observation of higher levels of soluble ACE2 in AD is in agreement with a previous report using a limited number of hippocampal samples of AD subjects (n = 13) compared to Controls (n = 5) [20]. Furthermore, preliminary human brain microarray data mentioned in a letter to the Editor also suggest a higher ACE2 expression levels in AD patients [51]. Although an association between SARS-CoV-2 infection and cognitive impairment has been previously evidenced at the population level [86] and hinted by genetic studies [28, 45], a significant correlation between ACE2 levels in the brain and cognitive scores has not been reported previously.

Several mechanisms could explain the higher levels of ACE2 in AD. Since old age increases the risk of infection with SARS-CoV-2 and of developing cognitive decline and AD, we could have expected an association between cerebral ACE2 levels and advanced age. However, no correlation between age and ACE2 could be evidenced here in both human and mouse samples, suggesting that changes in TBS-soluble ACE2 are not directly related to age but rather to AD pathology, as supported by the correlations observed with Aβ and tau pathologies in human subjects. This is consistent with health records data showing that dementia is associated with a higher risk for COVID-19, independently of age [86]. Second, the increase in ACE2 could be a consequence of the AD neurodegenerative process. Indeed, a recent network analysis suggested that AD and COVID-19 share defects in neuroinflammation and microvascular injury pathways [96]. Although we did not observe changes in murine ACE2 protein levels in the 3xTg-AD model, it should be reminded that such a mouse model displays an amount of Aβ and tau 1 to 3 orders of magnitude lower than what is typically found in an AD brain. A compensatory mechanism in response to AD neuropathology, including an increase in gene transcription, is consistent with the higher ACE2 mRNA expression measured in AD samples.

ACE2 is part of the renin angiotensin system (RAS), which regulates the vascular system in the whole body. An increase of cerebral ACE2 may impact the brain RAS, thereby affecting blood flow, arterial pressure, neuroinflammation and, consequently, brain function. Such a dysregulation of the RAS equilibrium in the brain could contribute to the aetiology of several neurodegenerative diseases, including AD [2, 87, 88]. For example, in a cohort study including community-dwelling older adults with mild to moderate AD, the use of ACE inhibitors (ACEi) was associated with a slower cognitive decline, independent from their antihypertensive effects [76]. ACEi and angiotensin II receptor blockers (ARBs) are also under investigation to improve cognitive impairment associated with AD [27, 30, 39, 71]. We did not detect any association with the use of drugs acting on ACE, such as ARBs or ACEi, and brain levels of ACE2, but the study was not designed for that purpose. However, it is important to note that the levels of ACE2 detected by immunoblotting may not directly inform on ACE2 activity. Indeed, a *postmortem* assessment with a fluorogenic assay instead showed a reduction of ACE2 enzymatic activity in AD [46]. Studies in animals indicate that pharmacological activation of ACE2 rather reduces hippocampal soluble Aβ and reverses cognitive impairment in the Tg2576 model of Aβ neuropathology [25]. A loss of function of cerebral ACE2 is expected to reduce levels of Ang1-7 and MAS/G receptor activity, leading to a decrease in anti-inflammatory, anti-oxidant, vasodilatory, and neuroprotective properties[42], which is undesirable in AD [30]. Imbalances in the brain RAS pathway have been shown to aggravate vascular pathology features associated with AD such as stroke or infarcts [30]. The increase in soluble ACE2 described here may involves a shift towards an inactive form of the enzyme, which may in turn translate in reduced ACE2-mediated response and defective brain RAS in the AD brain.

Another peculiar observation is the difference between ACE2 found in soluble fractions containing intracellular/extracellular proteins *versus* ACE2 retrieved in detergent-soluble fractions containing membrane-bound proteins. Overall, ACE2 in TBS-soluble fractions was higher in subjects with AD, while no such trend was observed with ACE2 from cell membranes. Moreover, the correlation between ACE2 and AD-relevant markers, most notably the pericyte markers PDGFRβ and others like ANPEP, differed significantly between the two fractions. The strong inverse association with TDP-43 pathology was also limited to detergent-soluble (membrane) ACE2. Previous studies did not distinguish TBS-soluble *versus* detergent-soluble ACE2 [20, 51]. Although ACE2 is generally considered a membrane protein, its actual attachment to the cytoplasm membrane is relatively weak. For example, the ACE2 ectodomain can be cleaved by ADAM17 or TMPRSS2 and released in the cytoplasm [37, 97]. Recent studies report that a decrease in active membrane-bound ACE2 due to ADAM17 and TMPRSS2 overactivation could be deleterious for SARS-CoV-2-infected patients [37, 65, 89]. However, we did not observe differences in mRNA and protein levels of TMPRSS2. Vascular ACE2 was also specifically measured in this study. Despite associations with cognitive scores and PDGFRβ levels, no significant difference was detected between groups, possibly due to the interindividual variability induced by the separation process. An intriguing possibility explaining the higher content in ACE2 specifically in the TBS fraction, as detected with an antibody targeting the N-terminal extracellular domain, could be an enhanced release of ACE2 from the membrane to the cytosol or the extracellular parenchyma in AD, also termed ACE2 shedding [37, 85]. Alternatively, abnormal intracellular trafficking could be involved, with unglycosylated ACE2 remaining trapped in the endoplasmic reticulum, leading to the intracellular accumulation of ACE2 instead of translocation to the cell surface [4, 6, 74]. Such a detachment of ACE2 from cell membranes may be a pathological phenomenon associated with AD, warranting further study. In any case, it would not directly facilitate SARS-CoV-2 entry into brain cells. As stated above, it would rather indicate a disturbance in ACE2 normal function and signalling, defective brain RAS and in turn, a fertile ground for SARS-CoV-2 aggravation in AD.

The present work also unveils additional information on the cellular localization of ACE2 in the human brain. Unlike in the mouse, where the enrichment in microvessels was evident, the detection of ACE2 in human brain capillaries became apparent only after microvascular fractionation. However, ACE2 was clearly present in neurons in human brain sections, corroborating Western blot results. Nonetheless, it should be noted that brains from mice were harvested quickly after transcardiac perfusion. On the other hand, human brain tissue underwent *premortem* and *postmortem* events, which may have affected ACE2 distribution and detection. In sum, the present data obtained using several different antibodies indicate that the cerebral distribution of ACE2 is less strictly vascular, more neuronal in the human brain compared to the mouse brain. At the very least, work related to human ACE2 but performed in mouse models should be interpreted with caution regarding their possible application to the brain RAS, AD and other neuropathologies, as well as central SARS-CoV-2 infection in humans.

## Conclusions

In summary, the present data show that an accumulation of the soluble form of ACE2 is associated with cognitive decline in individuals with a neuropathological diagnosis of AD. ACE2 levels were not influenced by age or biological sex. We also observed a strong association between soluble ACE2 levels and AD neuropathology, as well as pericyte loss. While such a rise in ACE2 could initially be interpreted as increased entry points for SARS-CoV-2 in the CNS, the observed shift toward a soluble form may more likely be an indication of defective brain RAS in AD. The search for molecular cues regulating ACE2 and the RAS in the brain may ultimately lead to the discovery of new therapeutics to prevent cognitive decline and AD.

### Supplementary Information

**Additional file 1**: Supplementary figures.

**Additional file 2**: Supplementary method: Isolation of murine brain microvessels.

## Data Availability

The datasets analysed during the current study available from the corresponding author Frédéric Calon on reasonable request. Data from the ROS can be requested at https://www.radc.rush.edu.

## Abbreviations

ACEi: Angiotension I converting enzyme inhibitors
ACE2: Angiotensin I converting enzyme 2
AD: Alzheimer’s disease
Ang1-7: Angiotensin-(1–7)
ANPEP: Aminopeptidase N
ARBs: Angiotensin II receptors blockers
BA: Brodmann area
BACE1: β-Secretase 1
BBB: Blood brain barrier
CD: Control diet
CNS: Central nervous system
COVID-19: Coronavirus disease 2019
DAPI: 4′,6-Diamidino-2-phenylindole
EHR: Electronic health records
FFPE: Formalin-fixed, paraffin-embedded
GAPDH: Glyceraldehyde-3-phosphate dehydrogenase
GWAS: Genome wide associations study
HRP: Horseradish peroxidase
HFD: High fat diet
MCI: Mild-cognitive impairment
NIA-AA: National Institute of AgingAlzheimer’s Association
NCI: No cognitive impairment
NeuN: Neuronal nuclear protein
NFT: Neurofibrillary tangle
NHS: Normal horse serum
O.D.: Optical density
PBS: Phosphate-buffered saline
pCAA: Parenchymal cerebral angiopathy amyloid
PDGFRβ: Platelet-derived growth factor receptor β
qPCR: Quantitative polymerase chain reaction
RAGE: Advanced glycosylation end product-specific receptor
RAS: Renin-angiotensin system
RBD: Receptor binding domain
mRNA: Messenger RNA
ROS: Religious order study
SARS-CoV-2: Severe acute respiratory syndrome CoronaVirus 2
SDS-PAGE: Sodium dodecyl sulphate‐polyacrylamide gel electrophoresis
SEM: Standard error of the mean
TBS: Tris-Buffered Saline
TDP-43: TAR DNA-binding protein 43
TMPRSS2: Transmembrane protease serine 2
