# Correction to: Higher angiotensin‑converting enzyme 2 (ACE2) levels in the brain of individuals with Alzheimer’s disease

**DOI:** 10.1186/s40478-023-01678-8

**Published:** 2023-11-01

**Authors:** Louise Reveret, Manon Leclerc, Vincent Emond, Cyntia Tremblay, Andréanne Loiselle, Philippe Bourassa, David A. Bennett, Sébastien S. Hébert, Frédéric Calon

**Affiliations:** 1 Faculty of Pharmacy, Laval University, Quebec, QC Canada; 2 CHU de Quebec Research Center, 2705, Boulevard Laurier, Room T2?05, Quebec, QC G1V 4G2 Canada; 3 Faculty of Medicine, Laval University, Quebec, QC Canada; 4 Rush Alzheimer’s Disease Center, Rush University Medical Center, Chicago, IL USA

**Correction to:** ***acta neuropathol commun*** **11, 159 (2023).**

10.1186/s40478-023-01647-1

Following publication of the original article [1], the authors identified an error in the author names of almost all authors. The given name and family name were erroneously transposed.

The author group has been updated above and the original article [1] has been corrected.
