# Supplementary material for: Higher angiotensin-converting enzyme 2 (ACE2) levels in the brain of individuals with Alzheimer’s disease
Source: Acta Neuropathol Commun. 2023 Oct 2;11:159. doi: 10.1186/s40478-023-01647-1 (PMC10544218; doi:10.1186/s40478-023-01647-1)
Supplement: Supplementary file 1 — Additional file 1: Supplementary figures. [file 40478_2023_1647_MOESM1_ESM.pdf]

Figure S1

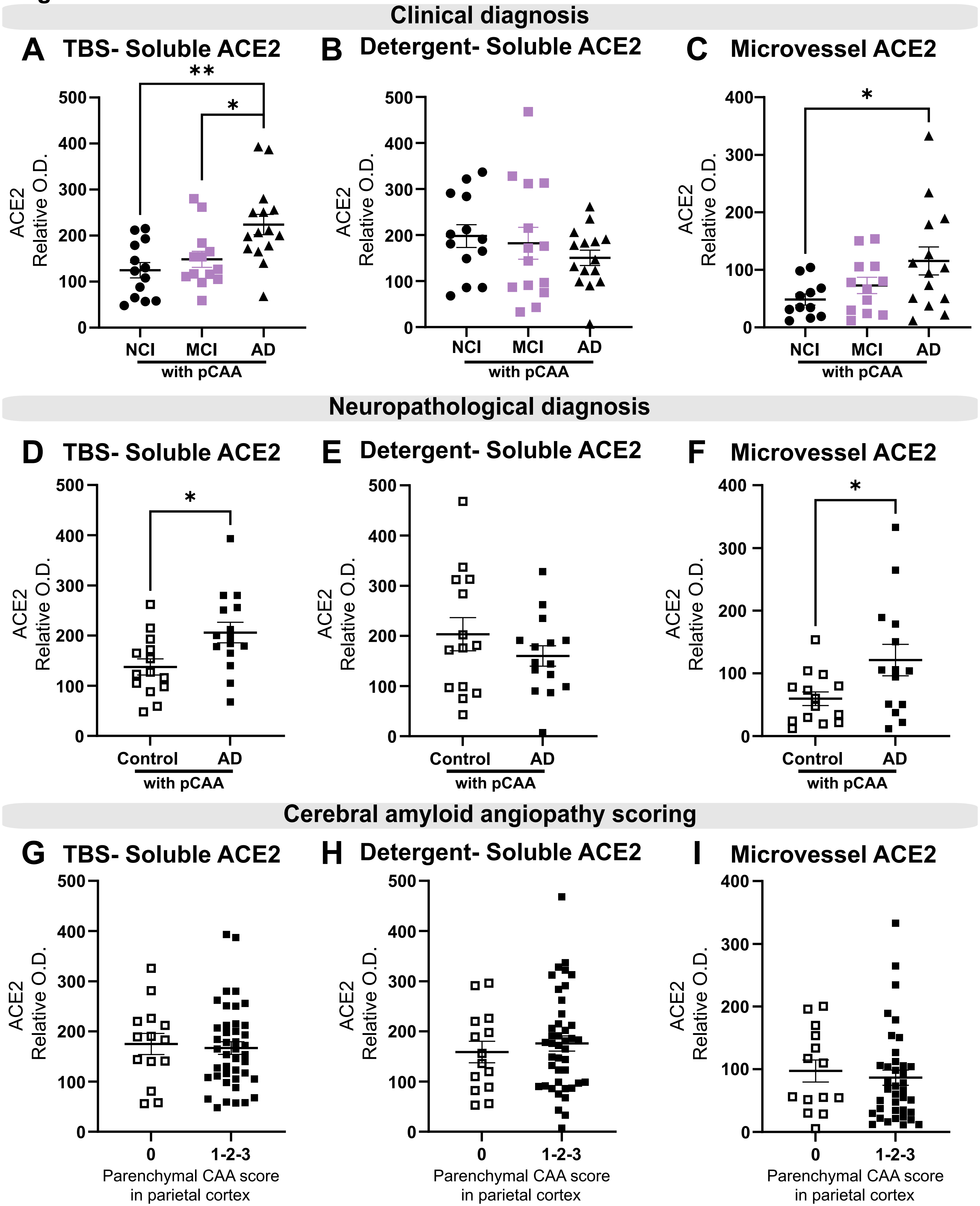
Clinical diagnosis
A TBS- Soluble ACE2
B Detergent- Soluble ACE2
C Microvessel ACE2
ACE2
Relative O.D.
NCI
MCI
AD
with pCAA
Neuropathological diagnosis
D TBS- Soluble ACE2
E Detergent- Soluble ACE2
F Microvessel ACE2
Control
AD
with pCAA
Cerebral amyloid angiopathy scoring
G TBS- Soluble ACE2
H Detergent- Soluble ACE2
I Microvessel ACE2
0
1-2-3
Parenchymal CAA score
in parietal cortex

Figure S2

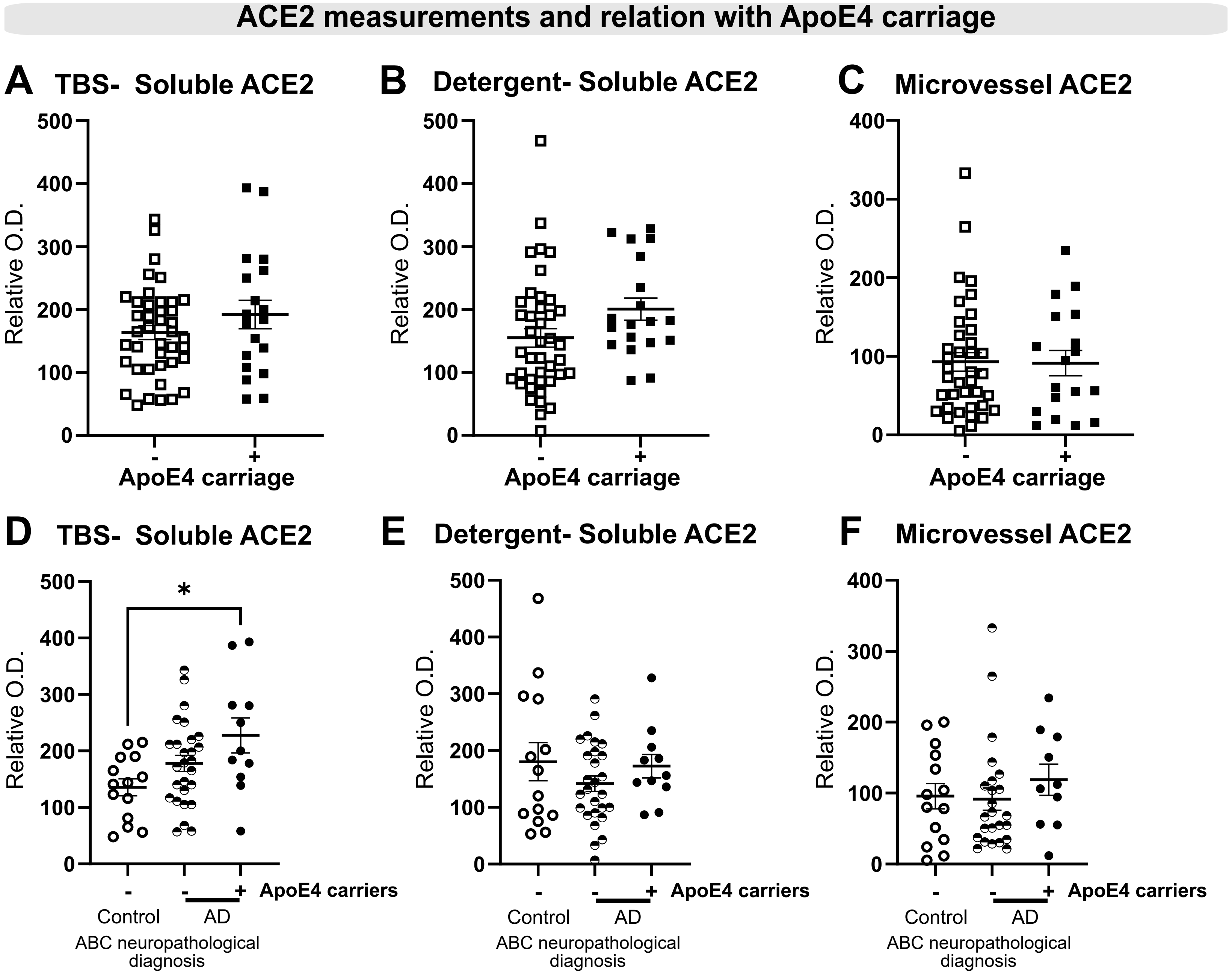
ACE2 measurements and relation with ApoE4 carriage
A
TBS- Soluble ACE2
B
Detergent- Soluble ACE2
C
Microvessel ACE2
Relative O.D.
ApoE4 carriage
-
+
D
TBS- Soluble ACE2
E
Detergent- Soluble ACE2
F
Microvessel ACE2
*
ApoE4 carriers
Control
AD
ABC neuropathological diagnosis

Figure S3

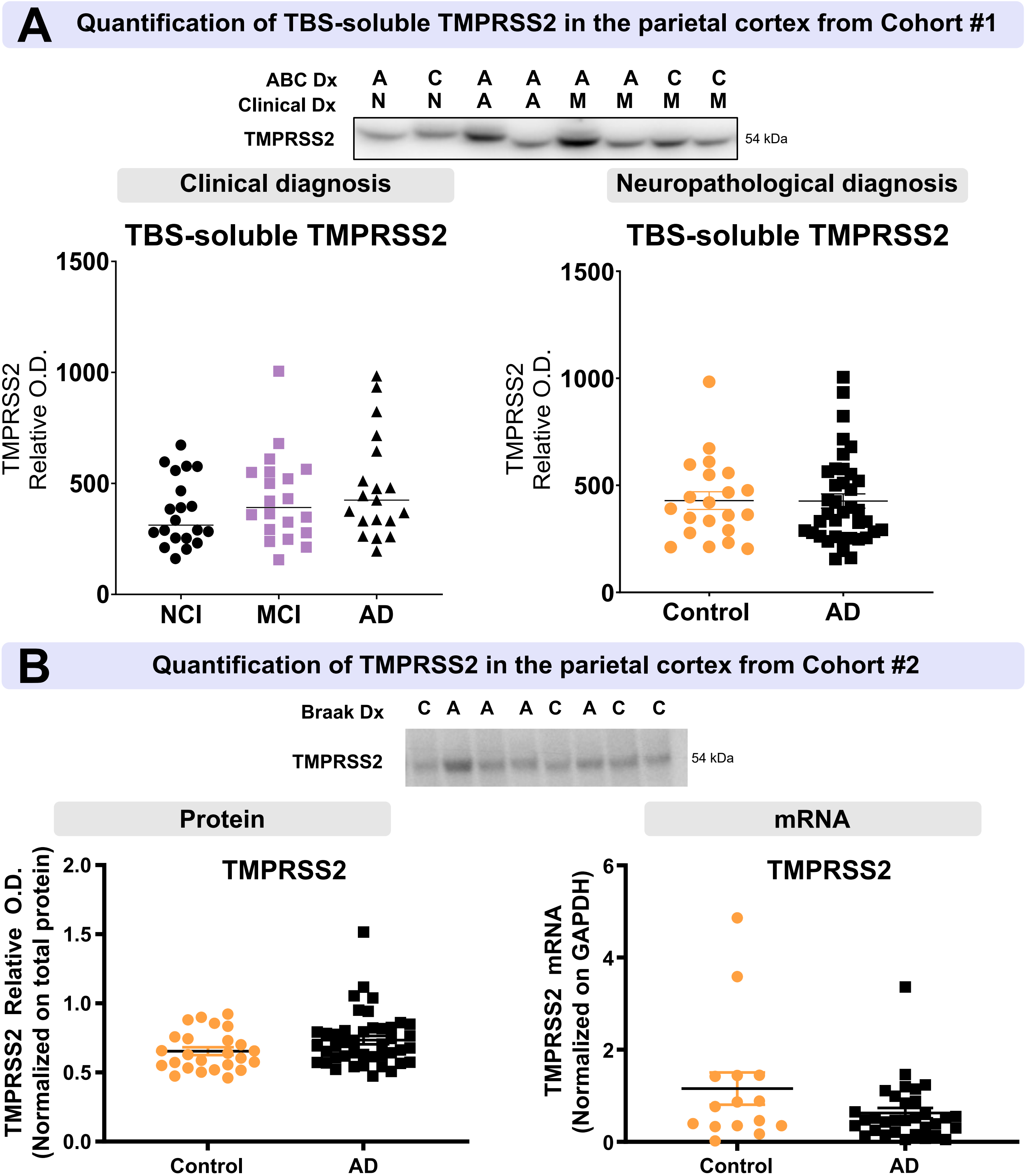

A
Quantification of TBS-soluble TMPRSS2 in the parietal cortex from Cohort #1
ABC Dx
A C A A A A C C
Clinical Dx
N N A A M M M M
TMPRSS2
54 kDa
Clinical diagnosis
Neuropathological diagnosis
TBS-soluble TMPRSS2
TMPRSS2 Relative O.D.
1500
1000
500
0
NCI
MCI
AD
TBS-soluble TMPRSS2
TMPRSS2 Relative O.D.
1500
1000
500
0
Control
AD
B
Quantification of TMPRSS2 in the parietal cortex from Cohort #2
Braak Dx
C A A A C A C C
TMPRSS2
54 kDa
Protein
mRNA
TMPRSS2
TMPRSS2 Relative O.D. (Normalized on total protein)
2.0
1.5
1.0
0.5
0.0
Control
AD
TMPRSS2
TMPRSS2 mRNA (Normalized on GAPDH)
6
4
2
0
Control
AD

Figure S4

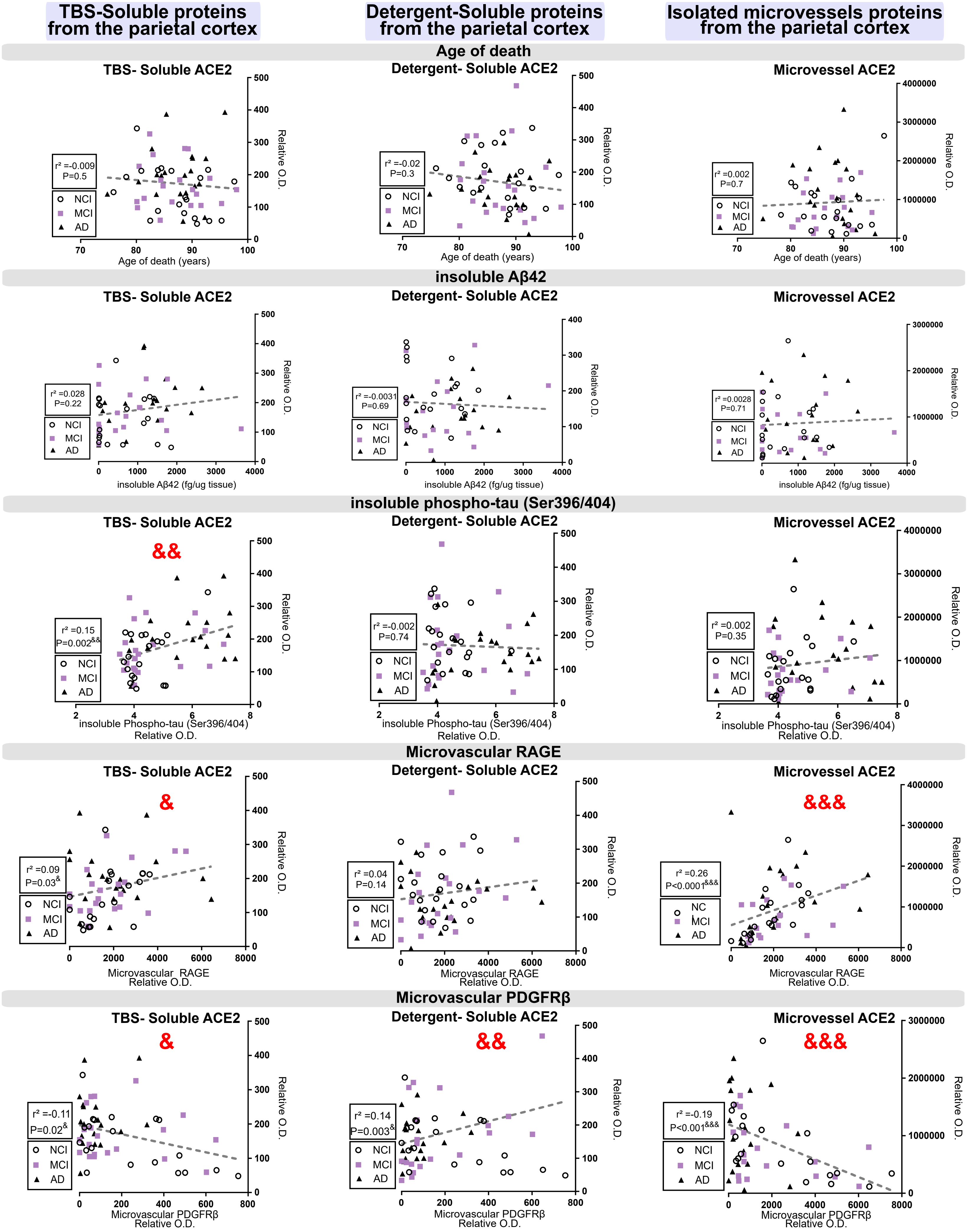
TBS-Soluble proteins from the parietal cortex
Detergent-Soluble proteins from the parietal cortex
Isolated microvessels proteins from the parietal cortex
Age of death
TBS- Soluble ACE2
r² =-0.009
P=0.5
Detergent- Soluble ACE2
r² =-0.02
P=0.3
Microvessel ACE2
r² =0.002
P=0.7
Age of death (years)
Relative O.D.
NCI
MCI
AD
insoluble Aβ42
TBS- Soluble ACE2
r² =0.028
P=0.22
Detergent- Soluble ACE2
r² =-0.0031
P=0.69
Microvessel ACE2
r² =0.0028
P=0.71
insoluble Aβ42 (fg/ug tissue)
insoluble phospho-tau (Ser396/404)
TBS- Soluble ACE2
&&
r² =0.15
P=0.002&&
Detergent- Soluble ACE2
r² =-0.002
P=0.74
Microvessel ACE2
r² =0.002
P=0.35
insoluble Phospho-tau (Ser396/404)
Relative O.D.
Microvascular RAGE
TBS- Soluble ACE2
&
r² =0.09
P=0.03&
Detergent- Soluble ACE2
r² =0.04
P=0.14
Microvessel ACE2
&&&
r² =0.26
P<0.0001&&&
NC
MCI
AD
Microvascular RAGE
Relative O.D.
Microvascular PDGFRβ
TBS- Soluble ACE2
&
r² =-0.11
P=0.02&
Detergent- Soluble ACE2
&&
r² =0.14
P=0.003&
Microvessel ACE2
&&&
r² =-0.19
P<0.001&&&
Microvascular PDGFRβ
Relative O.D.

Figure S5

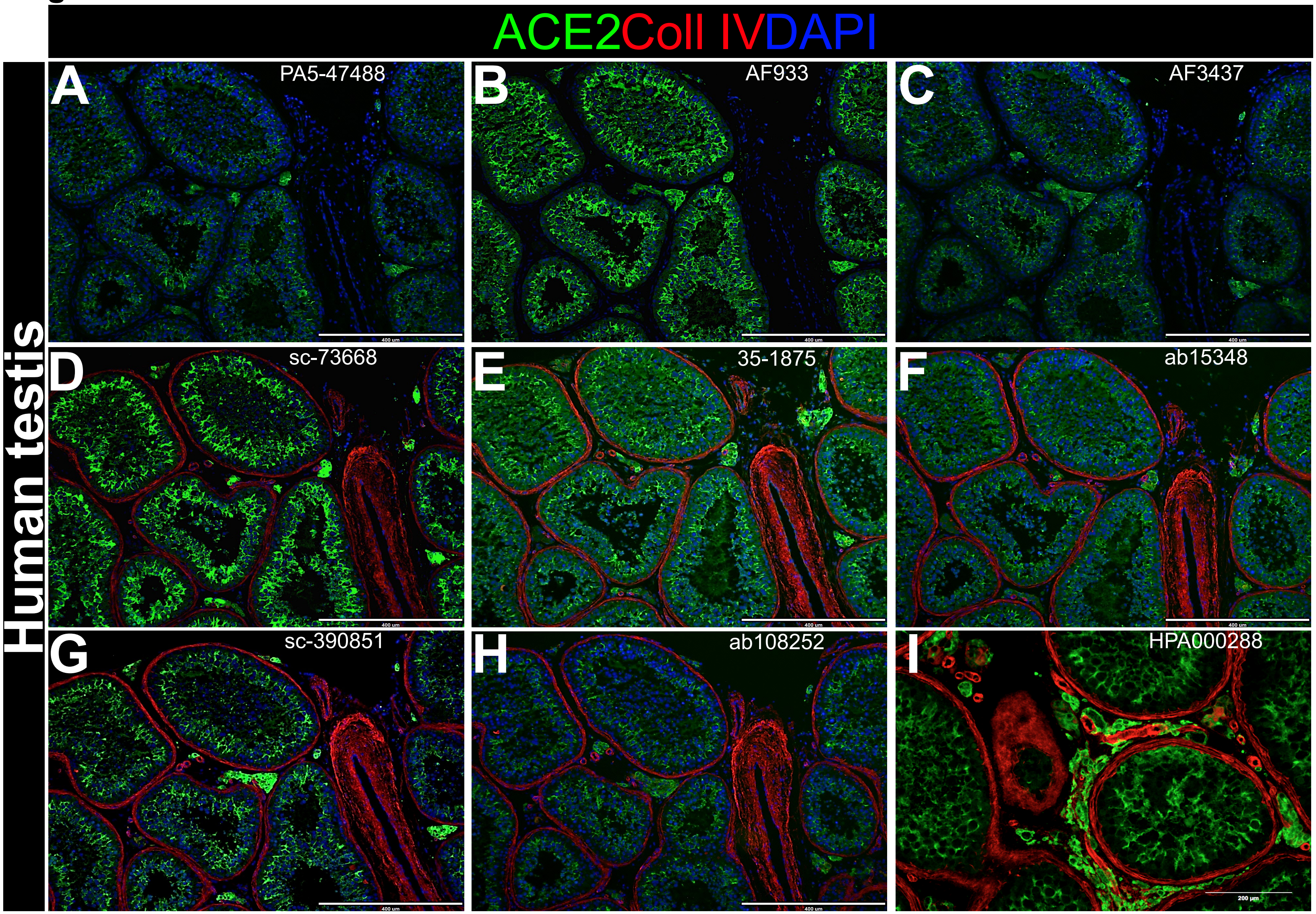
ACE2Coll IVDAPI
Human testis
A
PA5-47488
400 um
B
AF933
400 um
C
AF3437
400 um
D
sc-73668
400 um
E
35-1875
400 um
F
ab15348
400 um
G
sc-390851
400 um
H
ab108252
400 um
I
HPA000288
200 µm

Figure S6

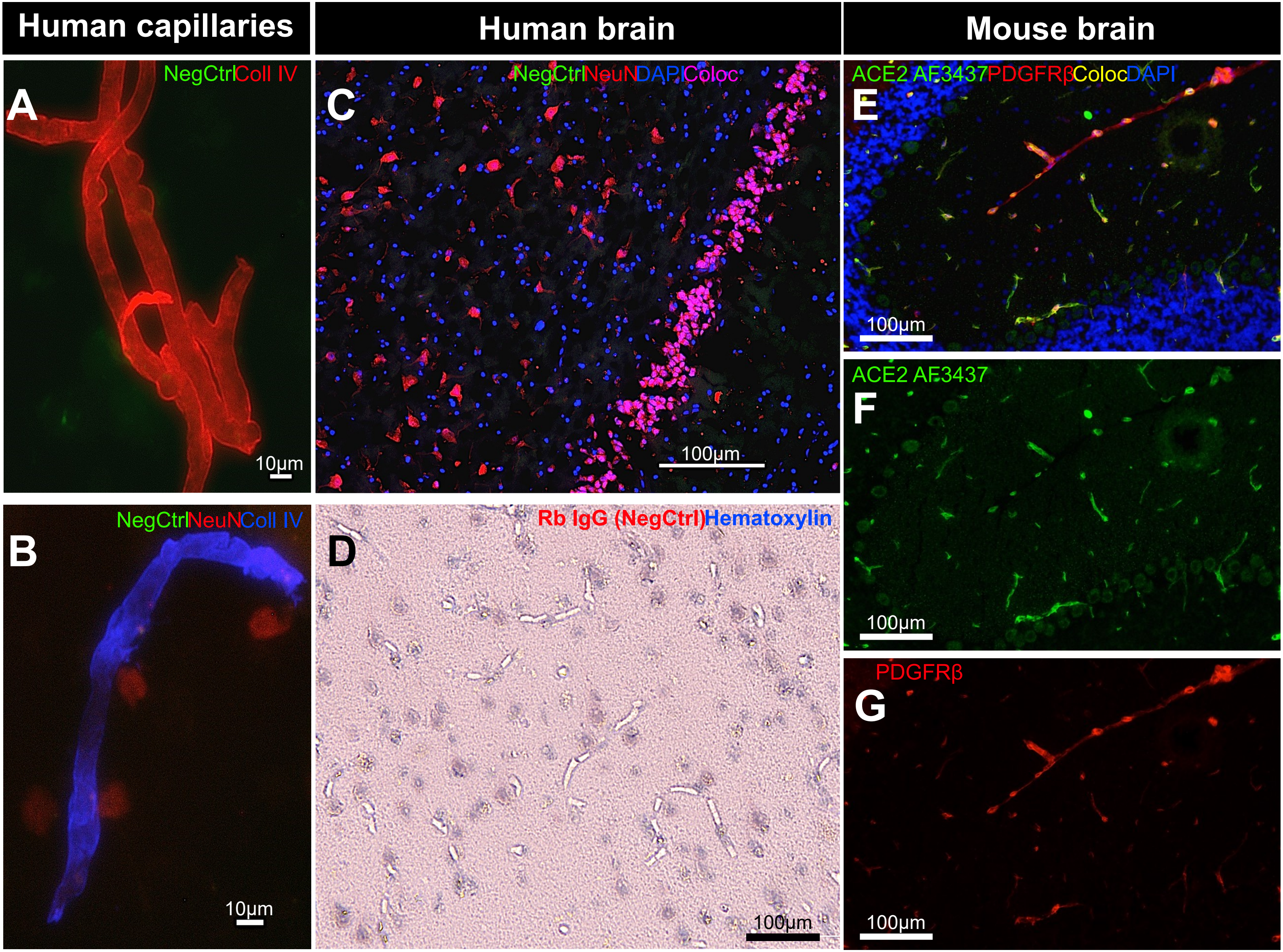

Human capillaries
Human brain
Mouse brain
A
NegCtrlColl IV
10µm
B
NegCtrlNeuNColl IV
10µm
C
NegCtrlNeuNDAPIColoc
100µm
D
Rb IgG (NegCtrl)Hematoxylin
100µm
E
ACE2 AF3437PDGFRβColocDAPI
100µm
F
ACE2 AF3437
100µm
G
PDGFRβ
100µm

Figure S7 **Measurements in human parietal cortex from Cohort#1**

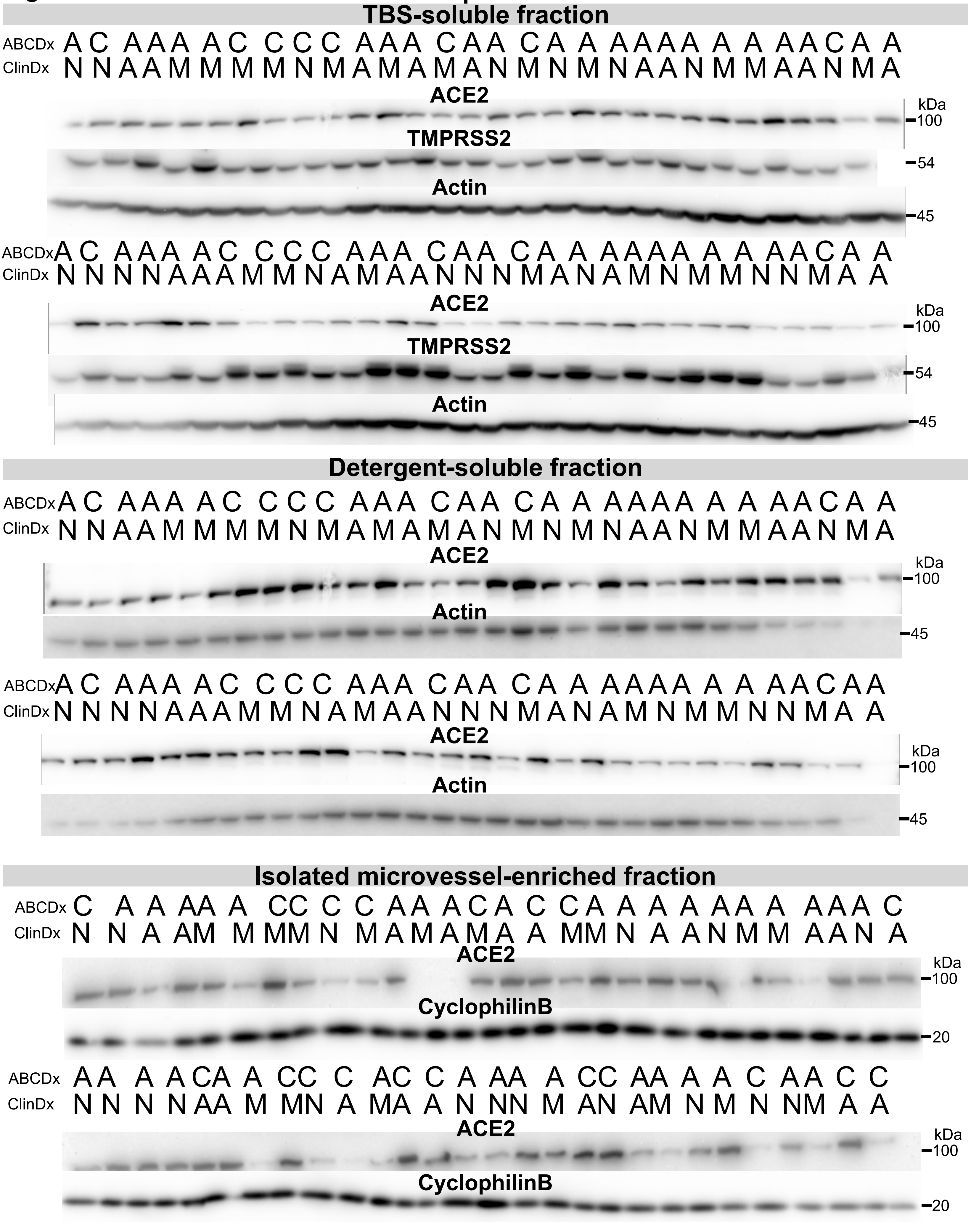

Figure S8

Measurements in human parietal cortex from Cohort#2

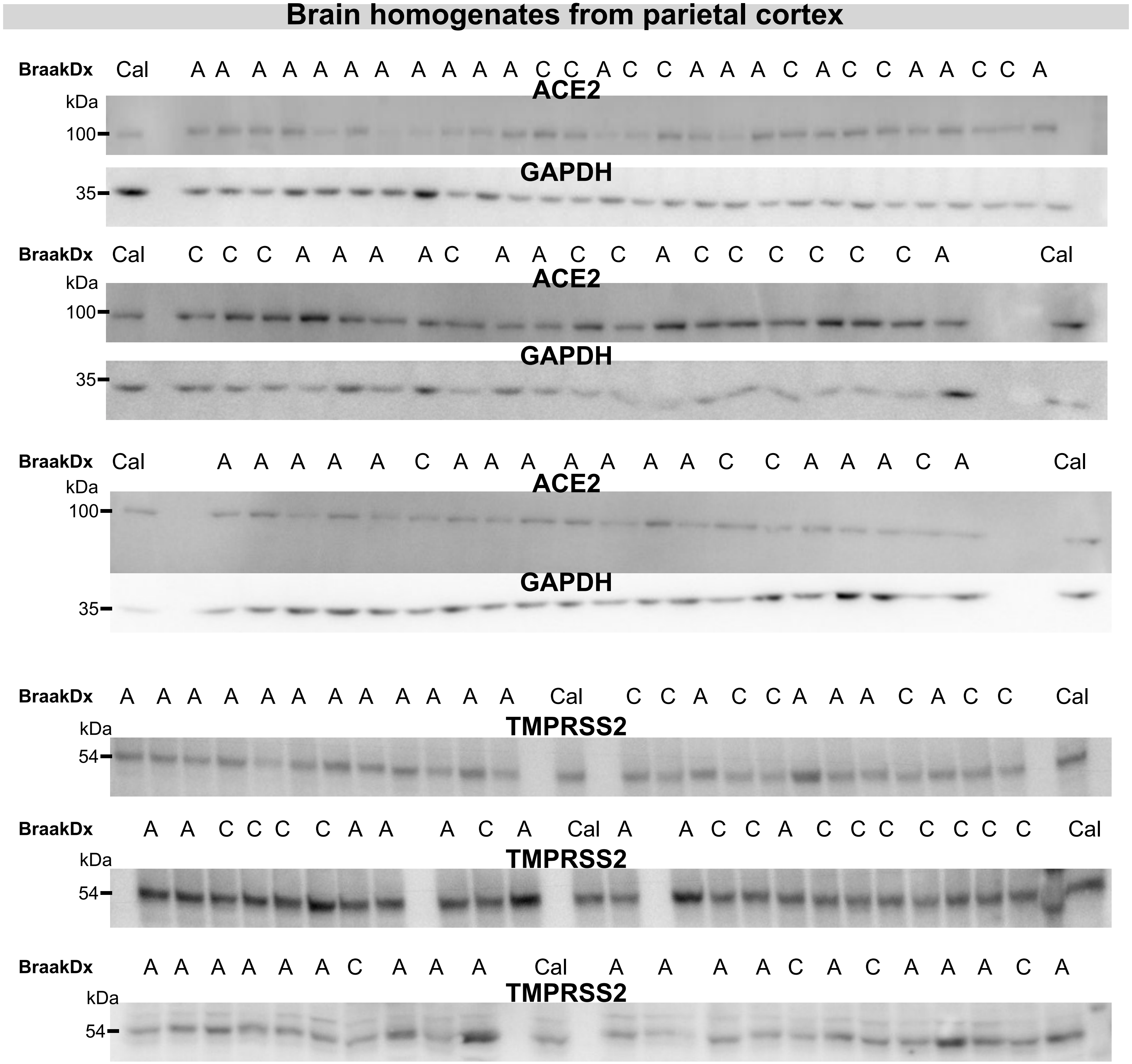

Figure S1: AD subjects with parenchymal CAA have higher soluble and microvessel ACE2. Individuals from Cohort #1 were grouped based on their ABC neuropathological diagnosis or clinical diagnosis and subdivided based on the presence of parenchymal CAA (pCAA) (A-F). Levels of soluble and microvessel ACE2 were higher in subjects with an AD neuropathological and clinical diagnosis with the presence of pCAA (A, C, D, F). Levels of detergent-soluble ACE2 did not change depending on the presence of pCAA. Participants were then divided according to their parenchymal CAA staging in the parietal cortex: meningeal and parenchymal vessels were assessed for amyloid deposition and scored from 0 to 4 [52](G, H, I). No statistical difference was observed in these comparisons. All samples, loaded in a random order, were run on the same immunoblot experiment for quantification. Data are represented as a scatterplot. Horizontal lines indicate mean ± SEM. Statistical analysis: two groups: unpaired t-test *p <0.05 or three groups: One-way ANOVA followed by a Tukey's multiple comparisons test *p<0.05, **p<0.01. *Abbreviations: ACE2, Angiotensin-Converting Enzyme 2; AD, Alzheimer's disease; MCI, mild cognitive impairment; NCI, healthy controls with no cognitive impairment; O.D., Optical density; pCAA, parenchymal cerebral amyloid angiopathy; SEM, standard error of the mean; TBS, Tris-Buffered Saline.*

Figure S2: AD ApoE4 carriers show higher levels of soluble ACE2. Participants of Cohort#1 were divided according to their ApoE4 allele carriage or to the neuropathological diagnosis, the AD group being further subdivided based on ApoE4. ApoE4 carriers with an AD neuropathological diagnosis had higher levels of TBS-soluble ACE2 when compared to control non carriers (D). Data are represented as a scatterplot. Horizontal lines indicate mean ± SEM. Statistical analysis: two groups: unpaired t-test, non-significant. Controls with ApoE4 were not included in for statistical analyses shown in D, E and F: One-way ANOVA followed by a Tukey's multiple comparisons test

*p<0.05. *Abbreviations: (-), ApoE4 non carrier; (+), ApoE4 carrier; ACE2, Angiotensin-Converting Enzyme 2; AD, Alzheimer's disease, O.D., Optical density; SEM, standard error of the mean; TBS, Tris-Buffered Saline.*

Figure S3: Levels of TMPRSS2 protein, which is employed by SARS-CoV-2 for Spike protein priming, are unchanged in AD individuals. (A) Levels of the TMPRSS2 protein were measured in Cohort #1: no difference was identified according to clinical or neuropathological (ABC) diagnosis. (B) In Cohort#2, TMRPSS protein and mRNA quantification did not reveal difference between control and AD. All samples, loaded in a random order, were run on the same immunoblot experiment for quantification. Examples were taken from the same immunoblot experiment, and consecutive bands loaded in random order are shown. Data are represented as a scatterplot. Horizontal lines indicate mean ± SEM. Statistical analysis: Ordinary on-way ANOVA or Mann-Whitney test, non-significant. *Abbreviations: A/AD, Alzheimer's disease; ABC Dx, ABC neuropathological diagnosis; Braak Dx, Braak staging diagnosis; C, control; Clinical Dx, clinical diagnosis; M/MCI, mild cognitive impairment; N/NCI, healthy controls with no cognitive impairment; O.D., Optical density; SEM, standard error of the mean; TBS, Tris-Buffered Saline; TMRPSS2, Transmembrane protease serine 2.*

Figure S4: Examples of correlation plots between ACE2 in the three protein fractions and age or AD-related proteins are shown. No significant correlation was established between ACE2 in all fractions tested and the age of death. No significant association were established between ACE2 and insoluble Aβ42. TBS-soluble ACE2 was positively correlated with phospho-tau AD2. Both TBS-soluble and microvessel ACE2 were positively associated with microvascular RAGE but negatively with microvascular PDGFRβ. Significant correlations are highlighted with the sign & in red. All proteins presented in these correlations were determined by Western blot analysis

except Aβ peptides, which were determined using ELISA. One outlier was removed from Aβ42 correlations plots. Coefficient of determination &p < 0.05, &&p < 0.01, &&&p < 0.001. *Abbreviations: ACE2, Angiotensin-Converting Enzyme 2; AD, Alzheimer's disease; MCI, mild cognitive impairment; NCI, healthy controls with no cognitive impairment; PDGFRβ, Platelet Derived Growth Factor Receptor Beta; RAGE, Receptor for Advanced Glycation Endproducts; Relative O.D., relative optical density; TBS, Tris-Buffered Saline.*

Figure S5: Human formalin-fixed paraffin-embedded testis sections were used as positive controls for ACE2 immunostaining, which is strong in this tissue. Antibodies used: (A) goat pAb #PA5-4788 from ThermoFisher, (B) goat pAb #AF933 from BioTechne, (C) goat pAb #AF3437 from BioTechne, (D) mouse mAb #sc-73668 from Santa Cruz, (E) rabbit pAb #35-1875 from Abeomics, (F) rb pAB #ab15348 from Abcam, (G) mouse mAb #sc-390851 from Santa Cruz, (H) rabbit mAb #ab108252 from Abcam and (I) rabbit pAb #HPA000288 from Atlas. Scale bars: 400 µm (A-H) and 200 µm (I). *Abbreviations: ACE2, Angiotensin-Converting Enzyme 2; Coll IV, Collagen IV; mAb, monoclonal antibody; pAb, polyclonal antibody.*

Figure S6: Negative controls for immunofluorescence and immunohistochemical experiments and channel split. (A) Human cerebrovascular fractions were immunostained for collagen IV (endothelial marker) in red with only secondary anti-rabbit antibodies (negative control for anti-ACE2, Figure 4BD) in green. (B) Human cerebrovascular fractions were immunostained for collagen IV in blue, as well as NeuN (neuronal marker) in red, with only secondary anti-rabbit antibodies in green (negative control for anti-ACE2, Figure 4C). (C) Fresh frozen human hippocampus sections were immunostained for NeuN in red, with only secondary anti-rabbit antibodies in green (negative control for anti-ACE2, Figure 5AB) and DAPI in blue. (D) Formalin-fixed paraffin-embedded parietal cortex were incubated with non-immune rabbit IgG (negative

control for anti-ACE2, Figure 5DE) revealed in red and counterstained with hematoxylin in blue. (E-G) Fresh frozen murine cerebellar sections were immunostained for ACE2 (AF3437) in green , PDGFRß in red and DAPI in blue. (E) Composite multichannel image. (F) Single channel image (ACE2). (G) Single channel image (PDGFRß). Scale bar: 10 or 100 µm. *Abbreviations: ACE2, Angiotensin-Converting Enzyme 2; Coll IV, Collagen IV; Coloc, Colocalization; IgG, immunoglobulin G; NegCtrl, negative control; NeuN, neuronal nuclear protein; Rb, rabbit.*

Figure S7: Western blots in human TBS-soluble, detergent-soluble and microvessel-enriched extracts from Cohort#1. The clinical and neuropathological diagnoses are given above each sample. *Abbreviations: A, Alzheimer's Disease; ABC Dx, ABC neuropathological diagnosis; ACE2, Angiotensin-Converting Enzyme 2; C, Control; ClinDx, Clinical diagnosis; M, mild cognitive impairment; N, healthy controls with no cognitive impairment; TBS, Tris-Buffered Saline; TMRPSS2, Transmembrane protease serine 2.*

Figure S8: Western blots in brain homogenates from Cohort#2. Diagnosis was determined using Braak staging. *Abbreviations: A, Alzheimer's Disease; ACE2, Angiotensin-Converting Enzyme 2; Braak Dx, Braak staging diagnosis; Cal, Calibrator; C, Control; GAPDH, Glyceraldehyde 3-phosphate dehydrogenase; TMRPSS2, Transmembrane protease serine 2.*
