# Supplementary material for: Higher angiotensin-converting enzyme 2 (ACE2) levels in the brain of individuals with Alzheimer’s disease
Source: Acta Neuropathol Commun. 2023 Oct 2;11:159. doi: 10.1186/s40478-023-01647-1 (PMC10544218; doi:10.1186/s40478-023-01647-1)
Supplement: Supplementary file 2 — Additional file 2: Supplementary method: Isolation of murine brain microvessels. [file 40478_2023_1647_MOESM2_ESM.docx]

**Supplementary method:**

*Isolation of murine brain microvessels*

The procedure used for isolation of murine brain microvessels has been reported in our previous work [14]. Nontransgenic and 3xTg-AD mice aged 6, 12 and 18 months were sacrificed with an intracardiac perfusion of ice-cold PBS containing 0.32 M sucrose and protease (SIGMA*FAST* Protease Inhibitor tablets, Sigma-Aldrich) and phosphatase (1 mM sodium pyrophosphate and 50 mM sodium fluoride) inhibitors, under deep anesthesia with ketamine/xylazine. The brains were immediately collected, and brainstem, cerebellum and meninges were removed. Murine brain samples were then chopped and frozen in 0.5 mL of HBSS containing 0.32 M sucrose and protease and phosphatase inhibitors (Bimake). For a milder freezing we used Mr. Frosty™ Freezing Container (Thermo Scientific). The microvessel enrichment procedure was then conducted as described for human samples. To validate the enrichment of mural cell markers, the microvessel-enriched and the microvessel-depleted fractions were compared to a total brain homogenate obtained from the homogenization of a whole hemisphere of a control mouse in the lysis buffer. Protein concentrations in all fractions were determined using the bicinchoninic acid assay (Thermo Fisher Scientific)
